# Vulnerable Nucleotide Pools and Genomic Instability in Yeast Strains with Deletion of the *ADE12* Gene Encoding for Adenylosuccinate Synthetase

**DOI:** 10.3390/ijms26083458

**Published:** 2025-04-08

**Authors:** Elena R. Tarakhovskaya, Yulia V. Andreychuk, Tatiana E. Bilova, Claudia Wiesner, Youri I. Pavlov, Elena I. Stepchenkova

**Affiliations:** 1 Vavilov Institute of General Genetics, Saint Petersburg Branch, Russian Academy of Sciences, 199034 Saint Petersburg, Russia; elena.tarakhovskaya@gmail.com (E.R.T.); yullinnabk@yandex.ru (Y.V.A.); 2 Department of Plant Physiology and Biochemistry, Faculty of Biology, Saint Petersburg State University, 199034 Saint Petersburg, Russia; bilova.tatiana@gmail.com; 3 Laboratory of Amyloid Biology, Saint Petersburg State University, 199034 Saint Petersburg, Russia; 4 Faculty of Chemistry and Mineralogy, Leipzig University, 04103 Leipzig, Germany; birkemeyer@chemie.uni-leipzig.de; 5 Eppley Institute for Research in Cancer, Fred and Pamela Buffett Cancer Center, University of Nebraska Medical Center, Omaha, NE 68198, USA; ypavlov@unmc.edu; 6 Department of Biochemistry and Molecular Biology, Microbiology and Pathology, Genetics Cell Biology and Anatomy, University of Nebraska Medical Center, Omaha, NE 68198, USA; 7 Department of Genetics and Biotechnology, Saint Petersburg State University, 199034 Saint Petersburg, Russia

**Keywords:** adenylosuccinate synthetase (AdSS), purine biosynthesis, metabolome, dNTP pool, mutagenesis, *Saccharomyces cerevisiae*

## Abstract

Adenylosuccinate synthetase (AdSS), encoded by the *ADE12* gene in yeast *Saccharomyces cerevisiae,* plays a critical role in purine biosynthesis, catalyzing the conversion of inosine 5′-monophosphate (IMP) and aspartic acid to adenylosuccinate, a substrate for the following adenosine 5′-monophosphate (AMP) synthesis step. Mutants lacking AdSS activity exhibit a range of pleiotropic phenotypes: slow growth, poor spore germination, accumulation, and secretion of inosine and hypoxanthine. We report new phenotypes of *ade12* mutants and explain their molecular mechanisms. A GC-MS analysis showed that *ade12* mutants have highly altered metabolite profiles: the accumulation of IMP leads to an impaired cellular energy metabolism, resulting in a dysregulation of key processes—the metabolism of nucleotides, carbohydrates, and amino acids. These metabolic perturbations explain the cell division arrest observed in *ade12* yeast strains. A slowed replication in *ade12* mutants, because of the insufficient availability of energy, nucleotides, and proteins, leads to the error-prone DNA polymerase ζ-dependent elevation of spontaneous mutagenesis, connecting multiple roles of AdSS in metabolism with genome stability control.

## 1. Introduction

**Figure 1:**
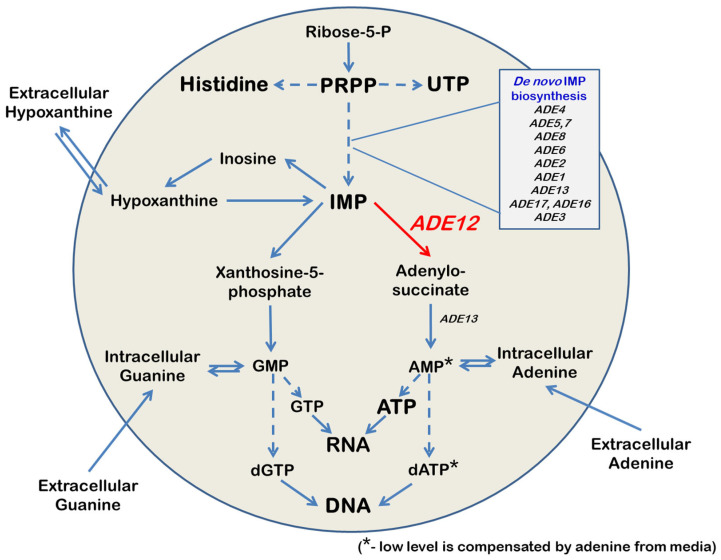
Scheme of the purine metabolism and routes of the extracellular nitrogenous base consumption. Direct reactions are presented as straight lines, and reactions involving several steps are presented as dashed lines. AMP—Adenosine 5′-monophosphate, ATP—Adenosine 5′-triphosphate, dATP—2′-Deoxyadenosine 5′-triphosphate, dGTP—2′-Deoxyguanosine 5′-triphosphate, GMP—Guanosine 5′-monophosphate, GTP—Guanosine 5′-triphosphate, IMP—inosine 5′-phosphate, PRPP—5-phosphoribosyl pyrophosphate, Ribose-5-P—*D*-Ribose 5-phosphate, and UTP—Uridine 5′-triphosphate.

Adenylosuccinate synthetase (AdSS) is critical in cellular metabolism (Figure 1). AdSS catalyzes the first committed step in the synthesis of adenosine 5′-monophosphate (AMP) [1,2,3], a precursor of nucleic acids, and signaling and energy-transmitting molecules. AdSS utilizes inosine 5′-monophosphate (IMP) as a substrate to synthesize adenylosuccinate. In the second step, adenylosuccinate lyase cleaves adenylosuccinate to produce AMP [1]. In addition to the de novo IMP biosynthesis [4,5], IMP can also be derived from extracellular purines through a branched salvage pathway [6,7]. Thus, IMP is a unique link between the salvage and de novo purine biosynthesis pathways (Figure 1).

The purine biosynthesis pathway is highly conserved in all living organisms, and AdSS was found in bacteria, fungi, plants, and animals [2,3,8,9,10]. While most organisms possess both de novo purine nucleotide biosynthesis and purine salvage pathways, certain pathogenic organisms, e.g., *Helicobacter pylori* [11] and *Leishmania gonovani* [12], rely exclusively on the purine salvage pathway for purine nucleotide synthesis. The development of AdSS inhibitors is considered to be a promising therapeutic strategy in the fight against antibiotic-resistant strains of these parasites [13,14]. In humans, mutations in the *ADSSL1* gene, which encodes a muscle-specific isoform of AdSS, are associated with an ultra-rare myopathy. The ADSSL1 myopathy was first described by Park and colleagues in 2016 in Korean patients suffering from distal muscle weakness [15]. Symptoms of the disease typically manifest during childhood and progress gradually with age. The affected patients experience distal muscle dysfunction and fatigue after mild physical exercise, which is later accompanied by facial muscle weakness. Cardiomyopathy and respiratory deficiency are also common in advanced disease stages, with death frequently caused by respiratory failure [16,17,18]. There have been 124 genetically diagnosed cases by 2024, mainly in Asian populations, but it is estimated that there could be about 4500 undiagnosed ADSSL1 myopathy patients worldwide [19].

Because of IMP’s central position in the purine metabolism pathway, it could be predicted that mutations in the genes encoding enzymes that control IMP utilization or biosynthesis will have pleotropic effects. This idea is undoubtedly true in the case of the *ADE12* gene encoding AdSS in yeast *Saccharomyces cerevisiae*. Like most mutants in the de novo purine biosynthesis pathway, yeast *ade12* mutants are auxotrophic for adenine [1,20]. All auxotrophic *ade* mutants, except *ade12*, can grow on a medium containing hypoxanthine as the sole purine source and utilize it via the purine salvage pathway. Yeast cells can utilize external hypoxanthine and convert it to IMP; however, AMP biosynthesis requires AdSS activity. Therefore, adenine, but not hypoxanthine, can compensate for the growth defect of the *ade12* mutants on minimal media [1,20]. Additionally, yeast *ade12* mutants exhibit slow growth even on complete media, and spores harboring the *ade12* mutation demonstrate poor germination rates. It is possible that *ade12* deletion blocks the production of AMP and, subsequently, ATP, leading to energy depletion and the slowdown of all energy-consuming processes required for cell growth, including DNA synthesis. Mutations affecting the upstream steps of purine metabolism rescue these defects [1,20]. It is also known that the *ade12* mutants accumulate hypoxanthine and inosine and excrete them into the medium [1]. Interestingly, in the food industry, IMP and GMP, combined with monosodium glutamate, increase the umami flavor. The performance of industrial strains of filamentous fungi *Ashbya gossypii*, producing IMP and GMP for umami flavor, is improved by AdSS inhibition, which increases the excretion of IMP and GMP into the medium [21]. In our previous experiments, we have found that *ade12* mutants are sensitive to mutagenic analogues of adenine (6-*N*-hydroxylaminopurine—HAP and 2-amino-6-hydroxylaminopurine—AHA) [22,23]. We propose that the toxic effect of *ADE12* deletion and sensitivity to HAP and AHA are caused by the elevated levels of IMP, which can presumably be converted into ribo- and deoxyribonucleosidetriphosphates. The excess ITP and dITP can saturate Ham1p—pyrophosphatase, an essential enzyme for the destruction of the nucleoside triphosphates of hypoxanthine [24,25], and HAP in nucleotide pools, leading to the frequent incorporation of mutagenic dHAPTP and dAHATP into DNA during replication [26,27].

The main goal of this study is to elucidate the mechanisms by which an AdSS deficiency leads to a decrease in the growth rate and an increase in mutation rates in *S. cerevisiae*, thereby contributing to a deeper understanding of purine biosynthesis and its broader biological consequences.

## 2. Results

### 2.1. Refined Phenotypes of the ade12 Null Mutant

**Figure 2:**
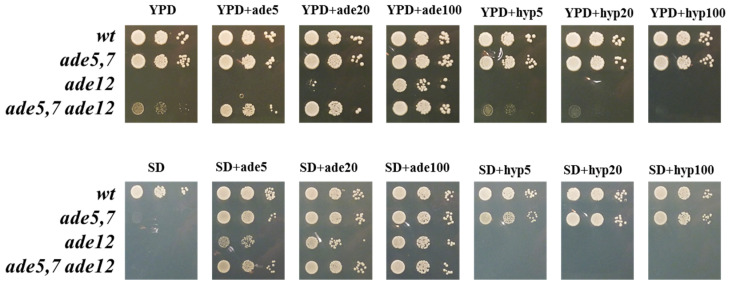
Deletion of the *ADE12* gene in yeast causes a growth defect that can be suppressed on medium containing extra adenine but not hypoxanthine. Ten-fold serial dilutions of the saturated yeast overnight cultures of WT, *ade5,7*, *ade12,* and *ade5,7 ade12* strains in YPDA were plated on YPD (upper panel) or SD (lower panel) containing adenine or hypoxanthine in different concentrations (0, 5, 20, or 100 µg/mL). Images were taken after 2 days of incubation at 30 °C.

We generated *ade12* mutants by replacing the *ADE12* gene with *TRP1* (see Materials and Methods, Table S1). The phenotypic characteristics of the newly derived *ade12* strains exhibited several differences from previous studies [1,20]. In particular, our *ade12* strains were unable to grow on the YPD medium and had growth defects on the synthetic (SD) dropout medium containing a low concentration of adenine (5 µg/mL) (Figure 2). Thus, the *ade12* null mutation is conditionally lethal. Previous studies have shown that hypoxanthine does not compensate *ade12*, and growth defects can be partially alleviated by the addition of supplemental adenine to the medium or through genetic modifications at earlier stages of IMP biosynthesis [1,20]. Double mutants carrying both *ade12* and *ade5,7* had significantly improved growth on the YPD medium compared to single *ade12* mutants (Figure 2), consistent with the literature [20]. To elucidate the difference between the YPD medium and synthetic dropout medium in the purine concentration, we measured the purine content in the yeast extract and peptone components used in preparing the YPD medium in our laboratory. The YPD medium contained 7 µg/mL adenine and 2 µg/mL hypoxanthine (Table S2). The concentration of adenine and hypoxanthine in the YPD medium alone cannot account for the significant differences in growth rates between YPD and SD medium (Figure 2). It is possible that other components in the YPD medium beyond the purine availability or slight differences in pH negatively impact the viability of *ade12* strains.

The *ade12* null strain unable to grow on the YPD produces YPD^+^ revertants with a relatively high frequency of 1.2 × 10^−5^. We picked up 567 independent YPD^+^ revertants and determined if they acquired additional mutations in purine metabolism genes using a genetic complementation test. We crossed YPD^+^ revertants to tester strains carrying mutations in one of the *ADE* genes (*ADE4*, *ADE5,7*, *ADE8*, *ADE6*, *ADE2, ADE1*, or *ADE3*); then, hybrids were examined for adenine auxotrophy (Table 1). Most of the YPD^+^ revertants exhibited an additional mutation in one of the *ADE* genes, and the frequency of these additional mutations correlated (r = 0.88, *p* = 0.02) with the gene length (excluding the *ADE3* gene) (Table 1). Around 20% of YPD^+^ revertants had suppressor mutations in another gene/other genes (Table 1). Interestingly, the *ade12* deletion strain clone in our laboratory, which was obtained from the Yeast Knockout (YKO) collection developed by the Saccharomyces Genome Deletion Project [28,29], carried an *ade4* mutation in addition to the *ade12*. This is consistent with our data (Table 1, Figure 2) and with the literature that additional mutations in *ADE* genes reduce the toxic effect of *ade12* [1,20]. It is likely that the strains considered single *ade12* mutants but growing on the YPD carry additional suppressor mutations.

### 2.2. Yeast ade12 Null Mutation Leads to a Moderate Increase in Pol ζ-Dependent Mutagenesis, Which Is Suppressed by a High Level of Adenine in the Medium

The relatively high frequency of the YPD^+^ revertants in the *ade12* strain led us to suspect an elevated mutability. We measured the frequency of direct mutations in the reporter *CAN1* gene in WT and *ade12* strains grown in a synthetic medium that contained 7 µg/mL adenine and 2 µg/mL hypoxanthine. The *ade12* mutant grew significantly worse than the WT strain (the relative survival of the *ade12* strain compared to WT was about 10%) and exhibited around five times the elevated mutant frequency (Table 2).

We measured the spontaneous mutation frequency in the double *ade5,7 ade12* mutant grown in the standard YPD medium and YPD supplemented with additional adenine (100 µg/mL) (YPDA). We have shown that, even in the presence of the *ade5,7* mutation, which suppresses the *ade12* lethality on YPD or SD with low concentrations of adenine, the *ade12* mutation increased the frequency of mutagenesis in the YPD medium without additional adenine (Table 3).

Additional adenine in the medium suppresses elevated mutagenesis in *ade5,7 ade12* strains (Table 3). In yeast, replication disturbance caused by mutations in the replicative DNA polymerase genes or by the addition of hydroxyurea, an inhibitor of ribonucleotide reductase, leads to a moderate increase in mutagenesis [30,31]. The increase is attributed to the recruitment of specialized DNA polymerase ζ (Pol ζ), whose catalytic subunit is encoded by the *REV3* gene. We hypothesized that, in *ade12* mutants, the increase in mutagenesis frequency is also associated with Pol ζ’s activity. We compared the mutagenesis frequency in *ade5,7 ade12* and the *ade5,7 ade12 rev3* strains to test this. The inactivation of the Pol ζ led to a reduction in mutagenesis to the level of the *ade5,7* strain, suggesting that mutations arising in the *ade12* background are introduced by Pol ζ. Thus, the genomic DNA becomes more accessible for the Pol ζ in *ade12* cells.

### 2.3. Overexpression of the ADE12 Is Not Mutagenic and Suppresses the Mutagenic Effect of HAP

We investigated the effect of the *ADE12* overexpression on spontaneous mutagenesis. We compared the frequency of the Can^r^ mutations in the *ade5,7* strains with either a multicopy plasmid (pRS425-ADE12) carrying the *ADE12* gene with its native promoter or the control vector pRS425. The spontaneous mutation frequency in the *CAN1* reporter gene did not differ significantly (Table 4). Thus, the overexpression of the *ADE12* is not mutagenic. Additionally, we assessed the influence of the *ADE12* overexpression on the sensitivity of the yeast strain to the mutagenic base analogue, HAP. Our findings revealed that the *ADE12* overexpression significantly reduced HAP-induced mutagenesis by nearly seven-fold (Table 4). These results are consistent with the previously obtained data indicating that AdSS is involved in the detoxification of HAP, as the deletion of *ADE12* leads to an increase in HAP-induced mutagenesis [22,23], while the overproduction of the protein ensures its reduction compared to the wild-type strain (Table 4). Thus, AdSS plays an active role in protecting the cell from the accumulation of the mutagenic deoxynucleoside triphosphate of HAP. It is probable that the cooperative action of several enzymes in the purine salvage pathway—specifically adenine deaminase (Aah1), guanine hypoxanthine phosphoribosyltransferase (Hpt1), and AdSS [22,23]—facilitates the rapid conversion of HAP to AMP within the cell. For instance, HAP is first converted to hypoxanthine by Aah1, after which Hpt1 catalyzes the synthesis of IMP by attaching a phosphoribose moiety to hypoxanthine. Subsequently, AdSS utilizes IMP as a substrate to synthesize AMP. Previous studies have demonstrated that *E. coli* AdSS can synthesize HAPMP from IMP and hydroxylamine (used instead of aspartate) and is capable of performing both the forward and reverse reactions [32]. Therefore, it is plausible that AdSS may also use HAPMP as a substrate, converting it to IMP, which is no longer mutagenic in yeast.

### 2.4. The ade12 Mutants Accumulate IMP Derivatives and Storage Carbohydrates and Lack Pyrimidine Nucleotide Precursors, Amino Acids, and Sugar-Phosphates

To investigate the potential metabolic pathways underlying the toxic effect of *ade12* mutation in *S. cerevisiae*, we characterized and compared the biochemical composition of the WT and mutant yeast cells incubated in the YPD and YPDA medium. The GC-MS-based metabolite profiling of *S. cerevisiae* revealed 360 individual analytes, of which 191 compounds were identified based on the coelution with authentic standards, retention index (RI) values, and spectral similarity, or structurally annotated to specific chemical classes by RI and spectral similarity to the MS-library data. The metabolite profiles were represented by 54 carbohydrates (sugars, polyols, and sugar acids), 45 amino acids and their derivatives, including 20 proteinogenic amino acids and 25 non-proteinogenic ones (sarcosine, 2-aminobutyric acid, pipecolic acid, β-alanine, ornithine, cysteinesulfinic acid, etc.), 27 organic acids (di- and tricarboxylic acids of TCA cycle, glyceric acid, benzoic acid, etc.), 19 fatty acids and their derivatives, 11 sterols (ergosterol, lanosterol, etc.), 10 nitrogenous bases, nucleosides, and their derivatives (adenine, uracil, hypoxanthine, adenosine, inosine, etc.), 7 amines (putrescine, spermidine, ethanolamine, etc.), 7 phenolic compounds (4-hydroxyphenylacetic acid, phenyllactic acid, phloroglucinol, etc.), and a miscellaneous group of metabolites including pantothenic acid, nicotinamide, phosphates, etc. (Table S3). The most abundant compounds in the metabolite profiles of the WT cells were glycerol, *myo*-inositol, trehalose, and several amino acids (glutamic acid, lysine, aspartic acid, and threonine). In the cells of *ade12* mutants, inosine was also one of the dominant analytes.

Regarding the detection of nucleosides in the GC-MS-based metabolite profiles, it is important to emphasize that these analytes may at least be partially derived from the corresponding nucleotides. Due to the hot flash evaporation of the liquid sample required for the GC-MS analysis, thermolabile nucleotide molecules may lose their phosphate moieties; those may also hydrolyze already during the sample preparation (in particular, derivatization) before chromatographic separation. Thus, changes in the nucleoside content may indicate changes in the amount of the corresponding nucleotides in the yeast cells. In particular, a high content of inosine in the samples of the *ade12* strain most probably suggests an accumulation of IMP in the mutant cells. The effect of the *ade12* mutation on the nucleotide pools was additionally measured using another approach (see Section 2.5).

**Figure 3:**
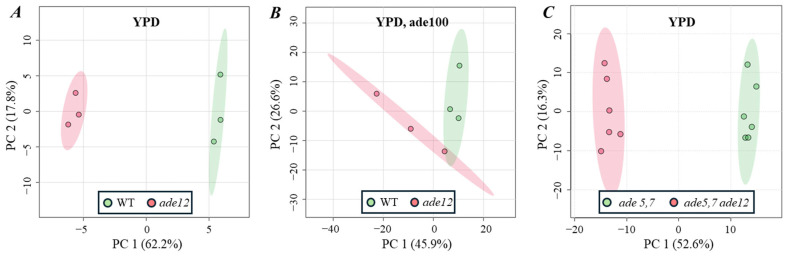
Sample scores for the first two principal components derived from PCA of the low-molecular-weight metabolite profiles of *S. cerevisiae* cells cultivated on pure YPD (**A**,**C**) or YPDA medium (**B**).

Differences between the metabolite profiles of the WT and mutant *S. cerevisiae* cells are clearly revealed by a principal component analysis (PCA), where the first two PCs explain 80% of the variance (Figure 3A). PC1 divides all the samples into two distinct groups, separating the WT cells and *ade12* mutants.

The contribution of specific metabolites to the *ade12*-dependent changes in the metabolite profiles of yeast was illustrated by the Volcano plot (Figure S1). Table 5 lists 63 identified or structurally annotated metabolites (excluding 51 unknowns) with significantly different abundances.

The *ade12* mutation dramatically changed the yeast metabolism (Table 5, Figure S1). The most affected groups of metabolites (fold change up to 7500) were nucleosides (in particular, inosine and its methylated derivative) and amino acids involved in the metabolism of pyrimidine nucleosides and nucleotides (4,5-dihydroorotic and orotic acids). Most of the metabolites showed a decreased abundance in the *ade12* mutants compared to the WT cells. The most prominent feature was a general drop in the content of amino acids (both proteinogenic and non-proteinogenic). The other downregulated groups of compounds were fatty acids, sugar phosphates, organic acids not involved in the TCA cycle, and several secondary metabolites (Table 5). The upregulated metabolites were presented by nucleoside inosine (presumably derived from IMP and, thus, reflecting IMP accumulation) and metabolically linked compounds (1-methylinosine, and hypoxanthine), organic acids (mainly TCA cycle intermediates), different carbohydrates (including the dominant compounds, trehalose and glycerol), three amino acids, ergosterol, and polyamine putrescine (Table 5).

At the next step of our study, we examined how the factors mitigating the toxic effect of *ade12* mutation (extra adenine supplementation or additional *ade5,7* mutation) might have influenced the metabolite profiles of *S. cerevisiae* cells. The addition of extra adenine (100 µg/mL) to the growth medium did not affect the metabolite profiles of the WT cells (Figure S2) but erased the *ade12*-dependent changes in the metabolism of yeast (Figure 3B, Table 6). In this case, samples of mutant cells had a much higher inter-group variance, and the list of compounds with significantly different abundances between the two compared groups included only five identified metabolites and one unknown analyte (Figure S3, Table 6). Most of the affected metabolites were the same as in the variant without adenine addition (Table 5), though with much lower fold change values. Notably, the analytes contributing the most to the difference between the metabolite profiles of WT and *ade12* without an adenine addition (inosine and 4,5-dihydroorotic acid) did not show significantly different abundances in the presence of extra adenine. We suggest that a higher concentration (compared to 100 µg/mL) of extracellular adenine would completely negate the changes in metabolite profiles of the yeast cells induced by the *ade12* mutation.

The metabolite profiles of the yeast cells bearing the *ade5,7* mutation differed considerably from the profiles of the WT cells (Figure S4). Moreover, PCA also showed a great difference between the *ade5,7* and *ade5,7 ade12* strains (Figure 3C). A deeper analysis of the specific compounds contributing to the *ade12*-dependent shift in the biochemical composition of the yeast cells on the background of the *ade5,7* mutation revealed 83 identified or structurally annotated metabolites (excluding unknowns) with significantly different abundances (*p* < 0.05, fold change ≥ 2) (Figure S5, Table S4). About 26% of affected metabolites were the same as those contributing to the *ade12*-dependent changes in the cells not bearing the *ade5,7* mutation: orotic acid, 2-aminobutyric acid, threonine, cysteine, β-alanine, glycerol-3-phosphate, ribose-5-phosphate, and lanosterol (downregulated); and glycerol, arabitol, mannitol, glyceric acid, α-ketoglutaric acid, putrescine, and hypoxanthine (upregulated) (Table 5 and Table S4). Notably, when accompanied by the *ade5,7* mutation, the *ade12* mutation did not result in the considerable accumulation of inosine and 1-methylinosine in the yeast cells. The most affected metabolites were uridine and orotic acid (downregulated, with fold changes of 125 and 46, respectively). Among the compounds accumulated in the *ade5,7 ade12* cells, different nitrogen-containing metabolites were conspicuous: polyamines and metabolically linked compounds (ornithine and arginine), nitrogen-rich amino acids (glutamine and histidine), and nitrogenous bases (hypoxanthine and uracil) (Table S4).

### 2.5. Inactivation of the ADE12 Gene Leads to a Dramatic Decrease in the NTP and dNTP Pools, Which Is Suppressed by the Adenine-Rich Media or by Blocking IMP Biosynthesis De Novo

**Figure 4:**
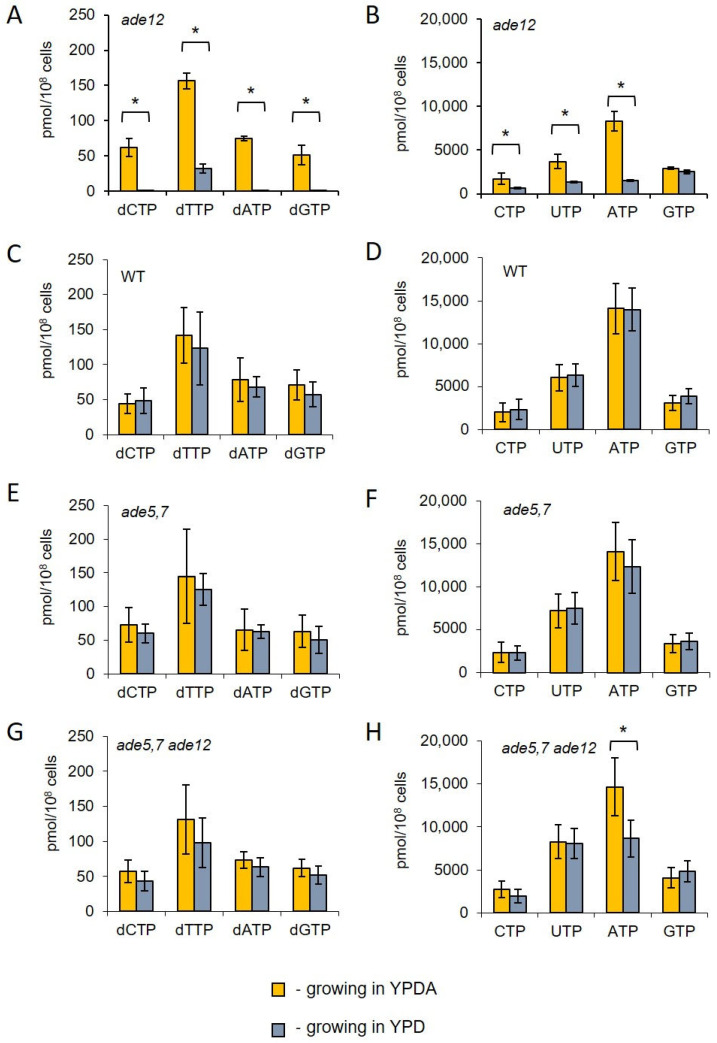
dNTP (**A**,**C**,**E**,**G**) and NTP (**B**,**D**,**F**,**H**) pools (pmol/10^8^ cells) in *ade12* (**A**,**B**), WT (**C**,**D**), *ade5,7* (**E**,**F**), and *ade5,7 ade12* (**G**,**H**) strains after 6 h of incubation in YPD. The strains were grown overnight in YPDA. The overnight cultures were diluted in the fresh YPD or YPDA medium until the optical density was 0.3–0.5. The samples were taken after 6 h of incubation in YPD or YPDA. * Statistically significant difference (*p*-value < 0.05), *t*-test.

**Figure 5:**
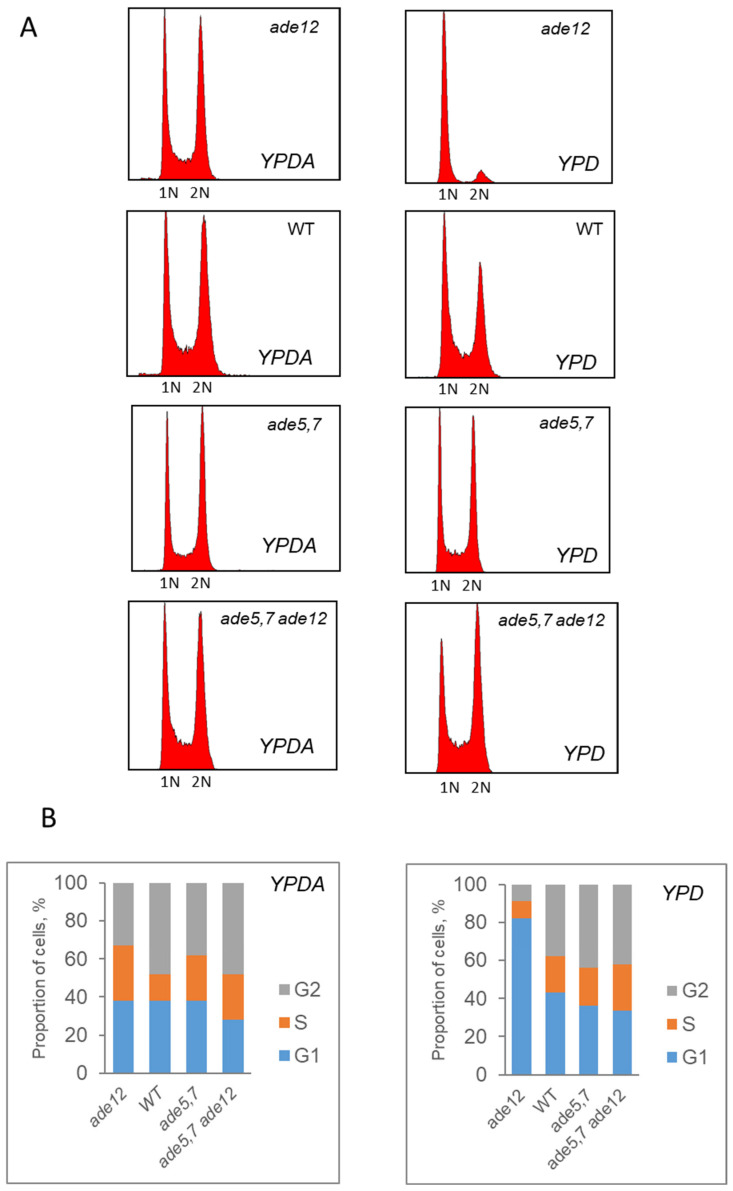
Analysis of the cell cycle progression by flow cytometry in the *ade12* mutants and control strains after 6 h of incubation in YPDA or YPD medium. DNA content in cells of *ade12*, *WT*, *ade5,7*, and *ade5,7 ade12* strains after incubation in YPDA or YPD medium (**A**). Proportion of cells in various stages of the cell cycle of *ade12*, *WT*, *ade5,7*, and *ade5,7 ade12* strains after incubation in YPDA or YPD medium (**B**).

The results obtained from the metabolomic analysis suggested that *ade12* mutants might have disturbed nucleotide pools. Specifically, there was a notable accumulation of inosine and its derivatives and a considerable decrease in the content of metabolites related to pyrimidine nucleotide synthesis. Therefore, we investigated the levels of NTPs and dNTPs in the cells of the WT, *ade5,7*, *ade12,* and *ade12 ade5,7* strains after 6 h of incubation in the YPD or YPDA medium. For this purpose, three cultures for each strain were first grown in the YPDA medium, and then cells were transferred to fresh YPD or YPDA medium and incubated further for up to 6 h. Aliquots of each culture were collected to evaluate the NTP and dNTP pools (Figure 4) and the cell cycle progression (Figure 5). We found that the *ade12* lethality in the YPD medium correlates with a severe decrease in intracellular nucleotide pools (Figure 4). After 6 h of incubation in YPD media, the dNTP pools in the *ade12* mutants decreased to almost undetectable levels of dCTP, dATP, and dGTP (Figure 4A). The content of CTP and UTP decreased three-fold, with the most dramatic decline (five-fold) for ATP (Figure 4B). These changes in the nucleotide pools make DNA synthesis impossible due to the absence of both building blocks (dNTP) and energy (ATP).

Unlike the *ade12* mutants, the WT (Figure 4C,D) and *ade5,7* (Figure 4E,F) strains did not demonstrate changes in the dNTP and NTP pools while growing in the YPDA and YPD media. Moreover, there was no statistically significant difference between the NTP pools of the double mutants *ade5,7 ade12* incubated in the presence of extra adenine (Figure 4 H) and the WT (Figure 4D) or *ade5,7* strains (Figure 4F). At the same time, the content of ATP in the *ade12 ade5,7* mutant grown in the YPD medium decreased by 30% (Figure 4H). The amounts of other NTPs (CTP, UTP, and GTP) and dNTPs in the *ade12 ade5,7* were unaffected (Figure 4G,H).

According to flow cytometry, *ade12* mutants exhibited a slowed cell cycle progression when incubated in the YPD medium (Figure 5). In the presence of extra adenine, the mutant strain did not differ from the WT, and both cultures contained the cells passing through the G1, S, and G2 phases of the cell cycle. However, after incubation in the YPD medium, the cultures of the *ade12* strain had an increased number of cells in the G1 phase, while the number of cells passing through the S and G2 phases decreased dramatically (Figure 5). This result is consistent with the main feature of the *ade12* phenotype: the inability to grow in the YPD medium lacking extra adenine.

## 3. Discussion

This study demonstrated that *ade12* mutants lacking AdSS cannot grow in a standard YPD medium and possess a mutator phenotype. Both manifestations are suppressed by the addition of adenine to the growth medium or by the genetic blocking of de novo IMP biosynthesis. To elucidate the molecular mechanisms underlying the phenotypes of the *ade12* mutation, we conducted genetic and biochemical analyses with a special focus on the nucleotide pools.

Flow cytometry data (Figure 5), in agreement with the results of the culture growth experiments (Figure 2), confirm that yeast cells bearing the *ade12* mutation cannot divide normally and do not progress through the cell cycle, stopping at the G1 phase. Similar phenotypes were also observed in the yeast strains with mutations in the other genes involved in AMP biosynthesis. For example, the *ade2-1* mutant (strain W303-1A) growing in a YPD medium without additional adenine supplementation had reduced cell division and budding rates, and a larger cell size [33]. Similarly, the *ade8* mutants grown in the medium lacking adenine (SD-ade) demonstrated a two-fold decrease in the budding rate compared to the control (SD+ade), an increase in cell size, and a predominance of cells with 1n DNA content indicative of G1-phase arrest [34].

We hypothesized that the most probable biochemical cause of cell division failure in the *ade12* strain is a deficiency of the key molecules maintaining this process. These molecules are nucleic acid constituents (nucleotides, nucleosides, and their derivatives), proteins, and energy-transferring compounds, such as ATP and NADH. The *ADE12*-encoded AdSS catalyzes one of the critical steps of purine nucleotide biosynthesis (Figure 1). Therefore, it is logical to assume that the mutant cells may encounter an overall reduction and imbalance of the nucleotide pools. Our findings indicate that, in *ade12* cells, NTP levels significantly decline when adenine is limited in the medium, and it is accompanied by an even more dramatic decrease in dNTP levels (Figure 4). The activity of the ribonucleotide reductase responsible for producing dNTPs is regulated by the NTP/dNTP ratio [35]. Consequently, as NTP levels decrease, dNTP production is also diminished, rendering DNA replication impossible.

The metabolomics data provide further insights into the *ade12*-dependent changes in cell biochemistry. Our results showed that *ade12* mutation leads not only to an imbalance of the nucleotide pools, but also to a general impairment of nitrogen exchange in the yeast cells. A decrease in the content of many amino acids (including both proteinogenic ones and those involved in nucleotide metabolism) and the accumulation of their precursors (such as α-ketoglutaric acid) imply a general inhibition of amino acid and, consequently, protein biosynthesis. Most of the nitrogen released due to this inhibition in the mutant cells is invested into forming IMP constituents and derivatives (inosine, methylinosine, and hypoxanthine) (Table 5). The accumulation of inosine and hypoxanthine in the yeast cells bearing the *ade12* mutation was seen earlier [1]. Notably, the author, similar to us, believed that this inosine was derived from IMP and, thus, was a manifest of IMP accumulation. In WT cells, the excessive accumulation of IMP is prevented by the feedback inhibition of purine biosynthesis by ATP and ADP [20,36]. However, this regulation fails in the *ade12* mutants lacking extracellular adenine [20].

**Figure 6:**
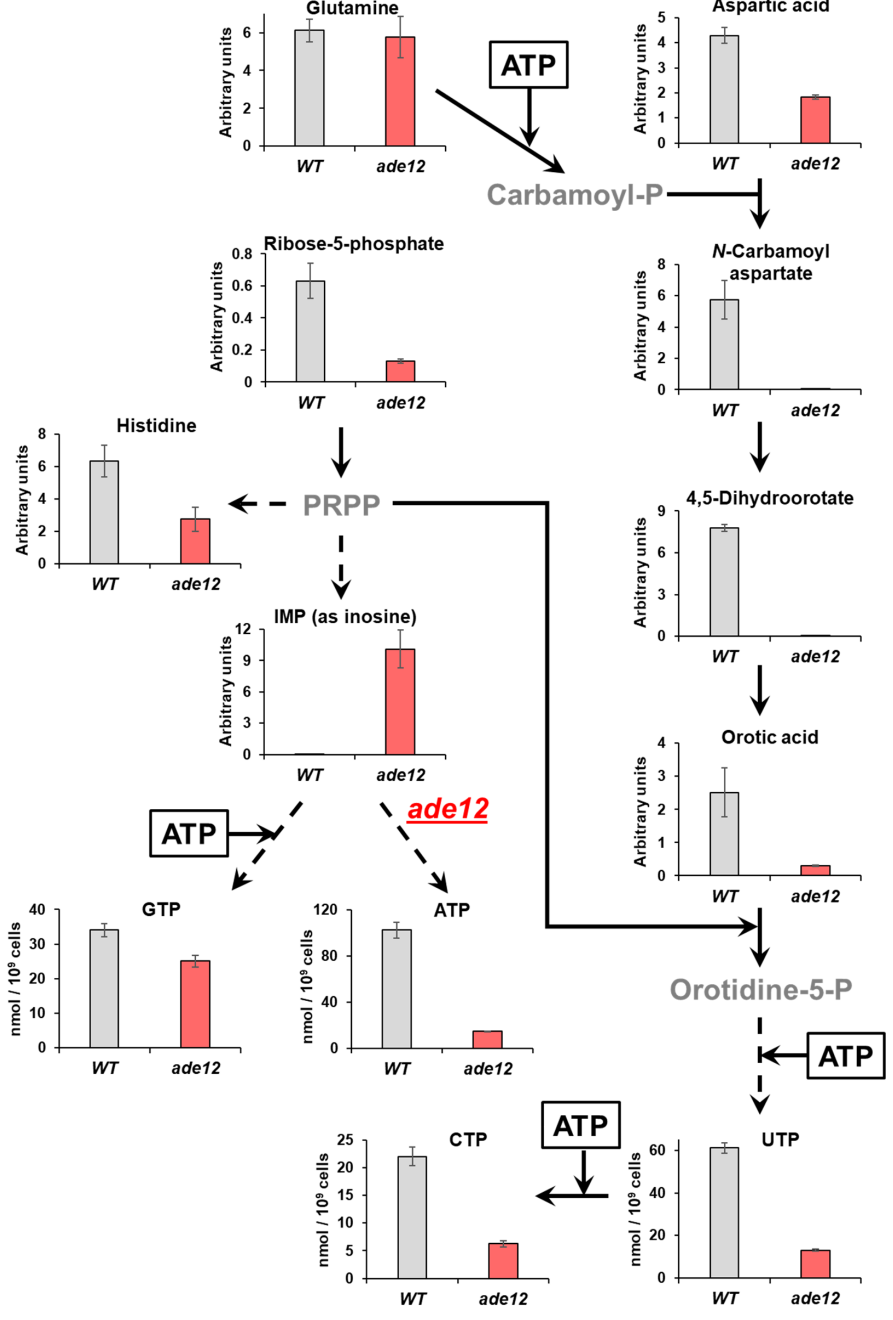
The *ade12*-dependent changes in the proportions of key intermediates in the pathways of de novo purine and pyrimidine nucleotide biosynthesis. Direct reactions are presented as straight lines, and reactions involving several steps are presented as dashed lines. Metabolites which were not determined are labeled in grey. IMP—inosine 5′-phosphate, and PRPP—5-phosphoribosyl pyrophosphate. IMP was measured as its derivative, inosine (see Section 2.4 for explanations). Bars represent the means ± standard deviation. Arbitrary units are normalized peak areas of extracted ion chromatograms.

Both the metabolite profiling and analysis of the nucleotide pools showed that the cells bearing a mutation in the purine biosynthesis pathway also exhibited considerable impairment in pyrimidine nucleotide biosynthesis. Thus, one of the most striking features of the metabolite profiles of *ade12* cells, along with inosine accumulation, was the three-orders-of-magnitude decrease in the content of 4,5-dihydroorotic acid, compared to the WT strain (Table 5, Figure 6). The purine and pyrimidine biosynthesis pathways are metabolically and energetically interconnected (Figure 6) [36], and we suggest that the main cause of the pyrimidine synthesis impairment in the *ade12* strain may be energy limitation. Indeed, the dramatic decrease in ATP content and a general decline in the amounts of sugar phosphates and nicotinamide (a NADH constituent) indicate that the *ade12* cells encounter severe energy deprivation (Figure 4 and Figure 6, Table 5). A similar drop in ATP and ADP content was shown earlier in the *ade8* mutants growing under adenine limitation [34]. We can see a decrease in the contents of the key intermediates of de novo pyrimidine biosynthesis at the next step after the first ATP-dependent reaction (formation of *N*-carbamoyl aspartate from glutamine via carbamoyl phosphate) (Figure 6). We could not detect carbamoyl phosphate in our samples, but its formation is likely suppressed in the *ade12* cells by an ATP shortage. This effect may be one of the reasons why glutamine is among the few amino acids whose content did not decline in the mutant cells. One more reason for the UTP and CTP decrease in the *ade12* strain may be the deficit of 5-phosphoribosyl pyrophosphate (PRPP), an intermediate enabling the metabolic crosstalk between the purine, pyrimidine, and histidine biosynthesis pathways (Figure 1) [36]. In the mutant cells, the main flux of PRPP is shifted towards the formation of IMP on account of the inhibition of the other pathway branches, supporting pyrimidine and histidine synthesis (Figure 6).

The other conspicuous feature of the metabolite profiles of the *ade12* cells is an enhanced accumulation of storage carbohydrates (trehalose, glycerol, and mannitol) compared to the WT strain (Table 5). Such an increase in carbon investment into metabolically relatively inert sugars and polyols is typical for non-dividing cells [37]. It may be explained by the impairment of nitrogen exchange and the inhibition of the biosynthesis of N-containing compounds (nucleic acids, proteins, and their monomers) in the *ade12* mutants with cell division arrest. Similar effects were seen for yeast cells under various stress conditions. Trehalose, the major soluble carbohydrate in yeast cells, not only serves as a storage compound but also plays a protective role under stress conditions [37]. Trehalose helps to maintain cellular integrity under heat, cold, desiccation, dehydration, and oxidation stresses primarily by preventing protein denaturation. Numerous studies, initiated by the pioneering work of Singer and Lindquist [38], have demonstrated that trehalose interacts with proteins and functions as a chemical chaperone, recovering protein aggregation and maintaining proteins in a partially folded state to facilitate their refolding by chaperones. Additionally, trehalose has been shown to prevent the amyloid aggregation of huntingtin [39] and amyloid beta [40]. Glycerol also plays a protective role in yeast: cells of *S. cerevisiae* rapidly accumulate glycerol when exposed to a highly osmolar medium to counteract dehydration [41,42]. We may conclude that the adenine limitation in *ade12* mutant leads to the activation of the protective mechanisms that typically serve to safeguard cells from adverse environmental conditions. According to the literature, mutations in the other *ADE* genes (*ade2* and *ade8*) also induce trehalose accumulation in the yeast cells, which is accompanied by a decrease in protein and nucleic acid content [33,34]. A similar phenotype has also been documented in yeast cells under the limitations of other compounds, such as uracil and leucine [43]. Our data on the changes in the carbohydrate composition in *ade12* cells are consistent with the findings from other studies indicating that the majority of yeast genes involved in purine biosynthesis (*ADE13*, *ADE1*, *ADE2*, *ADE4*, *ADE5,7*, *ADE6*, *ADE8,* and *ADE16*) are involved in the metabolism of carbon sources [44,45]. It has been shown that *ade13* mutants cannot grow in a complete medium containing glucose, but can grow in medium containing glycerol or ethanol as the sole carbon source. Additional mutations (*ade1*, *ade2*, *ade4,* and *ade5,7*) occurring at earlier stages of purine biosynthesis alleviate the conditional lethality associated with the *ade13* mutation [44]. Mutations in the *ADE2* gene result in the inability of yeast cells to utilize hypoxanthine as a purine source in a glycerol medium. Mutations in *ade4*, *ade5*, *ade8*, *ade6,* and *ade7* suppress this phenotype of the *ade2* mutation [45]. It has been shown that the expression of the *ADE16* gene, but not *ADE17*, depends on the type of carbon source in the medium. Specifically, the amount of Ade16p protein is higher in cells grown on non-fermentable carbon sources than in cells grown in the glucose medium [46]. Thus, the *ADE* genes, including *ADE12*, either directly regulate glycolysis or are involved in shaping cellular responses to the type of carbon source in the medium. The effect is explained by the need to coordinate the cell division with the nucleotide synthesis rates required for DNA replication and other cellular processes, depending on the quality of the carbon sources present in the environment [44]. The pivotal role of AdSS in coordinating purine and carbohydrate metabolism was supported by findings from a metabolite screening of endogenous factors that mediate glucose-induced insulin secretion in the rat insulinoma cell line 832/13. Glucose stimulation resulted in insulin secretion, decreased IMP levels, and increased adenylosuccinate, a product of AdSS [47].

Biochemical studies of the *ade12* cells grown in the presence of extra adenine or with the additional *ade5,7* mutation enabling the block of de novo IMP biosynthesis showed that both these factors effectively mitigate the most conspicuous *ade12*-dependent metabolic disorders, such as an IMP accumulation and 4,5-dihydroorotate decrease, as well as nucleotide pool imbalance (Table 6 and Table S4, Figure 4). Though the double *ade5,7 ade12* mutants still have a reduced ATP content when grown without the adenine addition, the difference is much smaller than that for the single *ade12* mutants (Figure 4). The mitigating effect of adenine supplementation may be explained by the restoration of the cell AMP pool due to the salvage pathway consuming extracellular adenine (Figure 1) [48,49,50]. The mechanism of the ameliorating influence of the *ade5,7* mutation is more complex, as this mutation itself considerably affects yeast cell metabolism (Figure S4). The metabolomic data imply that the main target of the *ade12*-dependent changes occurring on the background of the *ade5,7* mutation is again the nitrogen exchange, as double mutants specifically accumulate multiple nitrogen-rich metabolites, such as polyamines, arginine, and histidine (Table S4). However, this branch of our research goes beyond the scope of this paper and deserves a separate, more in-depth study, which is an ongoing plan in our laboratory.

Another question we addressed in the current research is the mechanism of the mutator phenotype of the *ade12* mutants. It is important to note that, in this study, we have shown, for the first time, that spontaneous mutagenesis is moderately increased in the strains bearing the *ade12* mutation; meanwhile, earlier, the *ade12* strain was also reported to have an increased mutation frequency induced by the mutagenic hypoxanthine analogues HAP and AHA [22,23]. Below, we discuss three non-contradictory explanations for the spontaneous mutator phenotype in the *ade12* strains.

First is the direct role of *ADE12* in maintaining the accuracy of genome replication. *S. cerevisiae* AdSS specifically binds to ss-DNA containing autonomous replication sequences (ARSs) [51]. This binding inhibits AdSS enzymatic activity and occurs only if AdSS is phosphorylated. However, the activity is specific for AdSS from *S. cerevisiae*, while AdSS from *E. coli* and *Dictiostelium discoideum* showed only 1% of the activity found in the yeast homologue. Thus, AdSS may regulate genome replication. If this is actually the case, then, in the absence of AdSS, the accuracy of replication and repair closely related to the stages of the cell cycle may decrease and lead to an increased level of spontaneous mutagenesis. There are several arguments against this hypothesis. Thus, ss-DNA binding activity was proven only in vitro, and it is unclear how the results correspond to AdSS properties in living cells. AdSS is located in the cytoplasm [52], thus making its DNA-binding activity irrelevant. Our findings do not support this mechanism. We have shown that elevated mutagenesis is suppressed in the *ade12* mutant in YPDA. Moreover, the overexpression of the *ADE12* gene does not affect the spontaneous mutation frequency. These results suggest that the level of spontaneous mutagenesis does not depend on the physical presence of AdSS in the origins of replication, but rather on AdSS’s role in purine metabolism.

A second assumption would be that the elevation of IMP in the cytoplasm of *ade12* mutants may promote the synthesis of dITP, which can then be incorporated into DNA, potentially leading to increased mutagenesis. However, the experimental data do not support this hypothesis, as the levels of inosine incorporation into DNA were reported to remain unchanged in the AdSS-deficient strains of both yeast and bacteria [53]. Moreover, it was shown in *E. coli* that dITP incorporation into DNA is non-mutagenic due to inosine’s strong pairing preference for cytosine [54]. This may be the case for yeast as well, as *S. cerevisiae* lacks inosine glycosylase due to the absence of a requirement to protect DNA from inosine incorporation.

A third idea is the indirect role of the missing AdSS in the increased mutation rate in the *ade12* strain. We have shown that the mutagenic effect observed in the *ade12* strain entirely depends on Pol ζ’s activity. Therefore, it appears that elevated IMP levels do not directly contribute to increased mutagenesis. In the *ade12* mutants grown in the YPD medium, dNTP pools and the ATP content are insufficient to maintain replication, resulting in cell cycle arrest. Under conditions of replication stalling, specialized DNA polymerase is more likely to be recruited for replication, similar to what occurs when yeast cells are incubated in a medium with hydroxyurea or during defective-replisome-induced mutagenesis (DRIM) [31]. This notion is supported by evidence that Pol ζ, unlike replicative polymerases, is active at low dNTP levels [55]. Interestingly, it was shown before that adenine starvation elevates the mutation rate in the yeast strains lacking the proofreading activity of DNA polymerase δ (*pol3-01*) [56]. Thus, through various mechanisms involving different DNA polymerases, adenine starvation in yeast can lead to an increased frequency of mutagenesis. The connections we have identified between nucleotide biosynthesis and elevated Pol ζ-dependent mutagenesis open up prospects for the discovery and development of nucleotide-analogue anticancer drugs [57].

We conclude that the low viability of *ade12* mutants in the standard YPD or synthetic medium low in adenine results from the impaired cellular energy metabolism, leading to a cascading dysregulation of key metabolic processes—the metabolism of nucleotides, carbohydrates, and amino acids. These metabolic perturbations may account for the cell division arrest observed in the *ade12* yeast strains. The mutagenic effect observed in *ade12* mutants appears to be a consequence of the slowed replication due to the insufficient energy, nucleotides, and proteins. Under these conditions, genomic DNA becomes more accessible to error-prone Pol ζ, increasing the likelihood of mutations. *ADSSL1* myopathy in humans presents a similar challenge regarding energy metabolism, where ATP deficiency is a key issue. The parallels between the metabolic dysregulation observed in *ade12* yeast mutants and human myopathies highlight the importance of understanding connections between energy metabolism and biosynthetic pathways in both yeast and higher organisms for the potential identification of therapeutic targets.

## 4. Materials and Methods

### 4.1. Yeast S. cerevisiae Strains, Plasmids, and Incubation Conditions

We used a series of yeast *S. cerevisiae* strains isogenic to the strain LAN201-ura3-Δ (*MAT*a *ade5-1 lys2-*Tn*5-13 trp1-289 his7-2 leu2-3,112 ura3-Δ*) [30]. First, we obtained T1 strain by replacing *ade5-1* mutation (C1158A) with WT allele of the *ADE5,7* gene. For that, we amplified 802 b.p. fragment of the *ADE5,7* gene using chromosomal DNA of the BY4742 strain as a template and two primers: ADE5,7-890 GTTAGAATATAATGTCAGATTCGG and ADE5,7-1668R GAATGTCAAGAGCACCAGTGGC. Then, we transformed LAN201-ura3-Δ strain with the PCR fragment and selected transformants on medium without adenine. The T1 strain was used to introduce disruptions of the *ADE5,7*, *ADE12,* and *REV3* genes. To make *ade5,7::kanMX* disruption, we transformed the T1 strain with the PCR fragment, obtained with the use of two primers ADE5,7-F GTGAAAGATGCCACCATACG and ADE5,7-R AAAGTGTCTTGCACCATACC and chromosomal DNA of *ade5,7* strain from Yeast Knockout collection [28,29]. To introduce *ADE12::TRP1* disruption, we first obtained PCR product using two primers AdeDISTRPL ATGGTTAACGTTGTATTGGGCTCCCAATGGGGTGATGAGGGTAAAGCAGAGCAGATTGTA and AdeDISTRPR GGTACCAACCCATTCAACAGGAACGCCAACAAAATCTTCAATATATTCGCATCTGTGCGG and pRS304 plasmid [58] as a template. Then, we transformed yeast cells with the PCR product and selected transformants on SD medium without tryptophan containing high amount (100 µg/mL) of adenine. To generate the *rev3::LEU2* disruption, we transformed yeast cells with the integrative pAM56 plasmid (constructed by A. Morrison), which was linearized with XbaI. All disruptions were validated by PCR and phenotypic analysis. To map suppressor mutations using a genetic complementation test, we employed a panel of tester strains carrying mutations in one of the *ADE* genes (*ADE4*, *ADE5,7*, *ADE8*, *ADE6*, *ADE2*, *ADE1*, or *ADE3*), obtained from the Peterhof genetic collection of yeast lines (Table S1).

To test effect of the *ADE12* overexpression, we cloned WT allele of the *ADE12* gene into pRS425 vector by BamHI and NotI restriction sites. For amplification of the WT copy of the *ADE12,* we used two primers ADE12_BAMH1_F TCGGGGATCCATGTCCCACTTACCTGTTCC and ADE12_NOT1_R GTACGCGGCCGCGGTGTTCGTTACATTGATTACTG, as well as chromosomal DNA of the BY4742 strain.

### 4.2. Media and Growth Conditions

Yeast cells were grown in standard YPD medium (0.5% Bacto^TM^ yeast extract (Becton, Dickenson and Company, Franklin Lakes, USA), 2% Bacto^TM^ peptone (Becton, Dickenson, and Company, Franklin Lakes, USA), 2% glucose), or in YPD medium with the addition of adenine (100 µg/mL) (YPDA), or in standard minimal synthetic medium (SD) (0.67% Yeast Nitrogen Base *w*/*o* amino acids (Becton, Dickenson and Company, Franklin Lakes, USA), 2% glucose, and amino acids, adenine, and uracil, required for growth of auxotrophic strains in standard concentrations) [51]. We also used YPD or SD medium supplemented with adenine or hypoxanthine at different concentrations (5, 20, or 100 µg/mL). Agar 2% was used to prepare the solid YPD, YPDA, or SD complete and dropout medium. SD medium containing 40 µg/mL of *L*-canavanine and all the supplements that are required for growth of auxotrophic strains was used for the selection of Can^r^ mutants. Frequencies of spontaneous Can^r^ mutations were determined as described [59].

### 4.3. Complementation Test

To verify whether the YPD^+^ revertants carry additional mutations in *de novo* adenine biosynthesis genes, we used a set of alpha mating-type tester strains, each carrying a mutation in one of the *ADE* genes (*ADE4*, *ADE5,7*, *ADE8*, *ADE6*, *ADE2*, *ADE1*, or *ADE3*) and the wild-type allele of the *ADE12* (Table S1). Independent clones of the *ade12* strain grown on the YPD medium were crossed with each tester strain. Then, we examined the ability of the resulting hybrids to grow on a medium lacking adenine. If the hybrid was prototrophic for adenine, it indicated that the tested YPD^+^ clone did not carry additional mutations in the *ADE* genes. If the hybrid of a YPD^+^ revertant was auxotrophic for adenine, the tested revertant carried a mutation allelic to the *ADE* mutation in the tester strain. After testing all YPD^+^ clones, we evaluated the frequency of mutations in each *ADE* gene in the *ade12* strains grown on the YPD medium.

### 4.4. GC-MS Analysis

Three to six independent cultures of each yeast strain were used for GC-MS analysis. Cultures of WT and *ade12* strains were first grown for two days in YPDA liquid medium. Then, two 1.5 mL aliquots were taken from each culture and washed three times with sterile distilled water. Cells from each aliquot were then diluted with 6 mL fresh YPD or YPDA and incubated for 4 h at 30 °C on a shaker. In case of *ade5,7* and *ade5,7 ade12* strains, overnight cultures in YPD medium at 30 °C were used. On completion of incubation, cells were harvested and washed three times with distilled water. After removal of all liquid, the cells were weighed and mixed with glass beads (200 µL) and 10 volumes of cold (−25 °C) methanol. The cells were then disrupted using a vortex mixer. After overnight extraction (4 °C), samples were centrifuged and aliquots of 100 µL of methanol extracts were transferred to clean 1.5 mL polypropylene Eppendorf tubes (VWR, Dresden, Germany) and vacuum-dried in the CentriVap vacuum concentrator system (Labconco, Kansas City, MO, USA) for subsequent analysis.

GC-MS analysis was carried out according to [60]. Briefly, vacuum-dried methanolic extracts were incubated by shaking in methoxyamine hydrochloride (Sigma-Aldrich Chemie GmbH, Taufkirchen, Germany) solution in pyridine and *N*,*O*-bis(trimethylsilyl)-trifluoroacetamide (Macherey-Nagel GmbH and Co KG, Düren, Germany). After derivatization, samples were transferred to glass vial micro-inserts and subjected to GC-MS analysis on a Trace GC Ultra gas chromatograph equipped with an A200S autosampler (CTC Analytics, Zwingen, Switzerland) and coupled with a MAT95 XP double-focusing sector field mass spectrometer (Thermo Electron, Bremen, Germany) with standard electron impact ionization (70 eV). Separation was accomplished on a DB-5MS Ultra Inert column (Agilent, Waldbronn, Germany; 30 m × 0.25 mm ID and 0.25 µm film) at 0.9 mL/min carrier gas flow (He 5.0 Alphagaz, Air Liquide, Düsseldorf, Germany) after splitless injection at 250 °C. Within each sequence, a mixture of alkanes (C10-C32) in hexane was measured for the calculation of Kovats retention indices. A mix of authentic standards containing 20 amino acids, 20 sugars and polyols, and 19 organic acids was co-spiked to confirm the identity of expected compounds.

Peak deconvolution was accomplished using AMDIS 2.66. The retention indices were automatically calculated using an AMDIS calibration file containing the batch retention times of each alkane. GMD (Golm metabolome database, GMD_20100614_VAR5_ALK, 24 September 2010), [61] and NIST14 (National Institute of Standards and Technology, Gaithersburg, MD, USA) were used for identification of the peaks based on spectra comparison. Experiments were carried out with 5 to 20 biological replicates (taken from different individuals). Quantitation of metabolites was performed by peak integration of the corresponding extracted ion chromatograms (*m*/*z* ± 0.5) for representative intense signals at specific retention times using Xcalibur 3.0.

MetaboAnalyst 6.0 web application (http://www.metaboanalyst.ca, accessed on 12 December 2024) was used for data processing and normalization procedures and creation of figures. The metabolomic data processing included peak area normalization to the median of all areas within the corresponding chromatogram, generalized logarithm transformation, and range data scaling (mean-centered and divided by the range of each variable). Normalized metabolomic data were subjected to a principal component analysis with Volcano plot representation. The results of univariate statistics were presented as mean ± standard deviation of 3–6 biological replicates. Fold change (FC) values in the abundance of individual metabolites and significance of differences (*p*-values) were estimated by Student’s *t*-test. The threshold values for FC and *p*-value presented in the tables and figures were 2 and 0.05, respectively. To assess the reliability of the FC in the relative levels of individual metabolites, false discovery rates (FDRs) at *p* ≤ 0.05 were estimated for all comparisons using the Benjamini–Hochberg method [62].

### 4.5. NTP and dNTP Pools Measurement

dNTP and NTP pools were measured as described in [63]. The cells were grown overnight in YPDA. The cultures were diluted to the OD = 0.2 in fresh liquid YPD media and incubated up to 6 h. The cells were collected and harvested by filtration through 25 mm White AAWP nitrocellulose filters (0.8 mm, Millipore AB, Solna, Sweden) at a density of 0.4 × 10^7^ to 0.5 × 10^7^ cells/mL, and NTPs and dNTPs were extracted in trichloroacetic acid and MgCl_2_ followed by extraction with a Freon–trioctylamine mix. dNTPs were separated from NTPs using boronate columns (Affigel 601, BioRad, Berkley, USA) and analyzed by HPLC on a LaChrom Elite UV detector (Hitachi, Tokyo, Japan) using a Partisphere SAX column (Hichrom, UK). For each strain, three independent clones were analyzed. Data are presented as mean ± standard deviation of 3 biological replicates.

### 4.6. Flow Cytometry

Cells from the growing cultures were analyzed for cell cycle progression and ploidy by staining of DNA with SYBR Green (Molecular Probes) as described [64] in a Becton Dickinson FC500.

## Figures and Tables

**Table 1:** Proportion of suppressor mutations among YPD^+^ revertants of the *ade12* strain.

Gene	Proportion of Revertants, %	Gene Length, b.p.
*ADE4*	11.1	1533
*ADE5,7*	22.2	2408
*ADE8*	1.5	645
*ADE6*	26.6	5077
*ADE2*	15.3	1716
*ADE1*	3.0	921
*ADE3*	0.5	2841
Genes outside the panel of tester strains	19.7	n/a

**Table 2:** Elevated frequency of Can^r^ mutants in *ade12* strains.

Strain	Median of Mutant Frequency × 10^−7^	95% Confidence Interval for Median
WT	9.6	6.9–16.0
*ade12*	50.2 *	32.0–102.5

* Statistically significant difference from WT strains (*p*-value < 0.001), Wilcoxon–Mann–Whitney test.

**Table 3:** The *rev3* mutation and additional adenine (YPDA) in YPD medium suppress mutability in *ade5,7 ade12* strain.

Strain	Medium	Median of Can^r^ Mutant Frequency × 10^−7^	95% Confidence Interval for Median
*ade5,7*	YPD	20.2	16.4–32.0
	YPDA	8.8	5.7–10.9
*ade5,7 rev3*	YPD	7.4	5.4–16.5
	YPDA	3.5	2.0–5.6
*ade5,7 ade12*	YPD	84.9 *	59.1–141.4
	YPDA	9.6	7.4–19.5
*ade5,7 ade12 rev3*	YPD	12.0	8.9–14.4
	YPDA	5.7	2.4–12.7

* Statistically significant difference from all other strains and conditions (*p*-value < 0.001), Wilcoxon–Mann–Whitney test.

**Table 4:** *ADE12* overexpression does not affect spontaneous and lowers HAP-induced mutagenesis.

Vector	HAP, µg/mL	Median of Can^r^ Mutant Frequency × 10^−7^	95% Confidence Interval for Median
pRS425	0	16.3	9.1–25.8
	5	3169.0 *	2761.2–3675.5
pRS425-ADE12	0	18.9	9.5–29.3
	5	478.9 *	286.6–560.6

* Statistically significant difference from all other strains and conditions (*p*-value < 0.001), Wilcoxon–Mann–Whitney test.

**Table 5:** The metabolites contributing to the *ade12*-dependent changes in the metabolite profiles of *S. cerevisiae*. The table includes identified or structurally annotated compounds having significantly (*p* < 0.05, FDR-adjusted) different abundances in the tested yeast strains (WT vs. *ade12*) with fold changes ≥ 2. The metabolites are grouped based on their chemical classes. The statistical analysis was performed using MetaboAnalyst 6.0 (http://www.metaboanalyst.ca, accessed on 27 January 2025).

Metabolite	Fold Change	Adjusted *p*-Value
Downregulated Metabolites		
Amino acids:		
4,5-Dihydroorotic acid	2883.3	0.001
Thiazolidine-4-carboxylic acid	11.4	0.002
2-Aminobutyric acid	9.3	<0.001
Orotic acid	8.4	0.005
Tryptophan	5.5	0.008
Phenylalanine	4.9	<0.001
β-Alanine	4.1	0.024
Ornithine	3.9	0.011
Leucine	3.5	0.001
Isoleucine	3.4	0.001
Homoserine	3.4	0.009
Serine	3.2	0.001
Arginine derivative	3.0	0.014
α-Aminoadipic acid	3.0	0.002
Glycine	2.9	0.037
Methionine sulfoxide	2.8	0.011
Cysteinesulfinic acid	2.7	0.010
Tyrosine	2.6	0.005
Aspartic acid	2.3	0.001
Histidine	2.3	0.031
Alanine	2.3	0.003
Threonine	2.2	0.040
Valine	2.2	0.003
Cysteine	2.0	0.013
Organic acids:		
Mevalonic acid	9.2	<0.001
2-Hydroxyisobutyric acid	6.5	0.005
2-Hydroxybutyric acid	2.6	0.004
Fatty acids and their derivatives:		
Lauric acid	4.9	0.001
Myristic acid	4.1	0.005
Pentadecanoic acid	2.7	0.036
1-Myristoylglycerol	2.6	0.007
Palmitelaidic acid	2.3	0.004
Sugar phosphates:		
Ribose-5-phosphate	4.8	0.002
Pentose phosphate RI2084	4.0	0.001
Glycerol-3-phosphate	3.9	<0.001
Miscellaneous:		
Adenosine	4.2	0.005
Nicotinamide	3.9	0.008
Lanosterol	2.8	0.005
2-Phenylethanol	2.2	0.018
**Upregulated Metabolites**		
Nucleosides and nitrogenous bases:		
Inosine	7505.1	<0.001
1-Methylinosine	42.0	0.004
5-Methylthioadenosine	4.0	0.010
Hypoxanthine	3.1	<0.001
Organic acids:		
α-Ketoglutaric acid	10.5	0.005
2-Hydroxyglutaric acid	8.0	0.001
Malic acid	4.2	0.001
Isocitric acid	3.2	0.010
Glyceric acid	2.6	0.002
Sugars and polyols:		
Glycerol	6.8	<0.001
Trisaccharide RI3564	3.4	0.007
Trehalose	3.2	0.001
Arabitol	2.5	0.002
Sugar RI3363	2.4	0.003
Trisaccharide RI3463	2.4	0.004
Disaccharide RI2976	2.2	0.013
Maltotriose	2.1	0.047
Disaccharide RI2994	2.1	0.016
Mannitol	2.1	0.034
Amino acids:		
Pipecolic acid	5.9	<0.001
Glutamic acid	3.1	0.010
Sarcosine	2.3	0.008
Miscellaneous:		
Ergosterol	10.0	0.005
Putrescine	3.6	0.037

**Table 6:** The metabolites contributing to the *ade12*-dependent changes in the metabolite profiles of *S. cerevisiae* grown in the YPDA medium. The table includes identified compounds having significantly (*p* < 0.05, FDR-adjusted) different abundances in the tested yeast strains (WT vs. *ade12*) with fold changes ≥ 2. The statistical analysis was performed using MetaboAnalyst 6.0 (http://www.metaboanalyst.ca, accessed on 27 January 2025).

Metabolite	Fold Change	Adjusted *p*-Value
**Downregulated metabolites**		
Glycerol-3-phosphate	3.2	0.005
Fumaric acid	2.9	0.005
2-Aminobutyric acid	2.9	0.010
**Upregulated metabolites**		
1-Methylinosine	18.1	0.005
Hypoxanthine	2.0	0.004

## Data Availability

Data are contained within the article and Supplementary Materials.

## Supplementary Materials

The following supporting information can be downloaded at: https://www.mdpi.com/article/10.3390/ijms26083458/s1.

## Abbreviations

The following abbreviations are used in this manuscript:

ADP	Adenosine 5′-diphosphate
AdSS	Adenylosuccinate synthetase
AHA	2-amino-6-*N*-hydroxylaminopurine
AMP	Adenosine 5′-monophosphate
ARS	Autonomous replication sequences
ATP	Adenosine 5′-triphosphate
b.p.	Base pairs
CTP	2′-Cytidine 5′-triphosphate
dATP	2′-Deoxyadenosine 5′-triphosphate
dCTP	2′-Deoxycytidine 5′-triphosphate
dGTP	2′-Deoxyguanosine 5′-triphosphate
dHAPTP	6-*N*-hydroxylaminopurine 2′-deoxynucleoside 5′-triphosphate
dITP	2′-Deoxyinosine 5′-triphosphate
dNTP	2′-Deoxynucleoside 5′-triphosphate
dTTP	2′-Deoxythymidine 5′-triphosphate
FDR	False discovery rate
GMP	Guanosine 5′-monophosphate
GS-MS	Gas chromatography–mass spectrometry
GTP	Guanosine 5′-triphosphate
HAP	6-*N*-hydroxylaminopurine
HAPMP	6-*N*-hydroxylaminopurine nucleoside 5′-monophosphate
HAPTP	6-*N*-hydroxylaminopurine nucleoside 5′-triphosphate
IMP	Inosine 5′-monophosphate
ITP	Inosine 5′-triphosphate
NADH	Nicotinamide-adenine dinucleotide
NTP	Nucleoside 5′-triphosphate
PCA	Principal component analysis
Pol ζ	DNA polymerase ζ
PRPP	5-phosphoribosyl pyrophosphate
RI	Retention index
Ribose-5-P	*D*-Ribose 5-phosphate
SD	Synthetic defined (yeast medium)
TCA	Tricarboxylic acid cycle
UTP	Uridine 5′-triphosphate
YKO	Yeast Knockout
YPD	Yeast extract, peptone, dextrose (yeast complete medium)
YPDA	Yeast extract, peptone, dextrose, adenine (yeast YPD medium, supplemented with 100 µg/mL of adenine)
