# Supplementary material for: Vulnerable Nucleotide Pools and Genomic Instability in Yeast Strains with Deletion of the ADE12 Gene Encoding for Adenylosuccinate Synthetase
Source: Int J Mol Sci. 2025 Apr 8;26(8):3458. doi: 10.3390/ijms26083458 (PMC12026682; doi:10.3390/ijms26083458)
Supplement: Supplementary file 1 [file ijms-26-03458-s001.zip › ijms-3545224-supplementary.pdf]

Supplementary Materials

Vulnerable Nucleotide Pools and Genomic Instability in Yeast Strains with Deletion of the *ADE12* Gene Encoding for Adenylosuccinate Synthetase

Elena R. Tarakhovskaya [1,2], Yulia V. Andreychuk [1,3], Tatiana E. Bilova [2], Claudia Wiesner [4], Youri I. Pavlov [5,6] and Elena I. Stepchenkova [1,3,7,*]

1 Vavilov Institute of General Genetics, Saint Petersburg Branch, Russian Academy of Sciences, 199034 Saint Petersburg, Russia; elena.tarakhovskaya@gmail.com (E.R.T.); yullinnabk@yandex.ru (Y.V.A.)
2 Department of Plant Physiology and Biochemistry, Faculty of Biology, Saint Petersburg State University, 199034 Saint Petersburg, Russia; bilova.tatiana@gmail.com
3 Laboratory of Amyloid Biology, Saint Petersburg State University, 199034 Saint Petersburg, Russia
4 Faculty of Chemistry and Mineralogy, Leipzig University, 04103 Leipzig, Germany; birkemeyer@chemie.uni-leipzig.de
5 Eppley Institute for Research in Cancer, Fred and Pamela Buffett Cancer Center, University of Nebraska Medical Center, Omaha, NE 68198, USA; ypavlov@unmc.edu
6 Department of Biochemistry and Molecular Biology, Microbiology and Pathology, Genetics Cell Biology and Anatomy, University of Nebraska Medical Center, Omaha, NE 68198, USA
7 Department of Genetics and Biotechnology, Saint Petersburg State University, 199034 Saint Petersburg, Russia
* Correspondence: stepchenkova@gmail.com

Supplementary Figures and Tables

Table S1. Strains of *Saccharomyces cerevisiae* used in this work.

Strain	Genotype	Source
LAN201-ura3-Δ	*MATa ade5-1 lys2*-Tn*5-13 trp1-289 his7-2 leu2-3,112 ura3-Δ*	[26]
T1	*MATa lys2*-Tn*5-13 trp1-289 his7-2 leu2-3,112 ura3-Δ*	This work
T1-a5,7	*MATa ade5,7::kanMX lys2*-Tn*5-13 trp1-289 his7-2 leu2-3,112 ura3-Δ*	This work
T1-a12	*MATa ade12::TRP1 lys2*-Tn*5-13 trp1-289 his7-2 leu2-3,112 ura3-Δ*	This work
T1-r3	*MATa rev3::LEU2 lys2*-Tn*5-13 trp1-289 his7-2 leu2-3,112 ura3-Δ*	This work
T1-a5,7,12	*MATa ade5,7::kanMX ade12::TRP1 lys2*-Tn*5-13 trp1-289 his7-2 leu2-3,112 ura3-Δ*	This work
T1-a5,7-r3	*MATa ade5,7::kanMX rev3::LEU2 lys2*-Tn*5-13 trp1-289 his7-2 leu2-3,112 ura3-Δ*	This work
T1-a5,7,12-r3	*MATa ade5,7::kanMX ade12::TRP1 rev3::LEU2 lys2*-Tn*5-13 trp1-289 his7-2 leu2-3,112 ura3-Δ*	This work
BY4742	*MATα his3Δ1 leu2Δ0 lys2Δ0 ura3Δ0*	[24,25]
α,ade4HR	*MATα ade4 ura1*	Provided by
α,ade5HR	*MATα ade5 ura1*	A.M. Zehnov
8B-799	*MATα ade7-23 lys9-14*	
4V-P117	*MATα ade8-Δ lys9-14*	(Peterhof Genetic

7A-885	*MATα ade6-8 arg4*	Yeast Collection)
jB101	*MATα ade2 lys9*	
8A-P3532	*MATα ade1-14 his7-1 met13-A1*	
3V-P109	*MATα ade3-Δ lys9-14*	
35B-P137	*MATα ade5,7-Δ lys3-Δ*	

Table S2. The amount of purine nitrogenous bases* in yeast extract and peptone used to prepare 1 ml of YPD.

Purine source	Adenine, µg	Hypoxanthine, µg
Yeast extract (5 mg)	6.7±0.4	1.9±0.2
Peptone (20 mg)	0.37±0.03	0.09±0.004
YPD (1 ml)	7.1	2.1

*Content of nitrogenous bases in the water solutions of yeast extract and peptone was measured by GC-MS analysis using the authentic standards of adenine and hypoxanthine (Sigma-Aldrich, Taufkirchen, Germany). The procedure of sample preparation and analysis is described in Materials and Methods (Section 4.3).

Table S3. Metabolites detected by GC-MS analysis in the wild type and mutant strains of *Saccharomyces cerevisiae*. The table contains 360 individual compounds, presented by one or several chromatographic peaks.

No.	Analyte[a]	Derivatization groups[b]	RT (min)	RI	Quantification, $m/z \pm 0.5$	Library, match factor[c]
1	Alanine peak 1	2TMS	10.01	1119.9	218	NIST (786), ASL
2	Glycine peak 1	2TMS	10.44	1138.1	204	NIST (886), ASL
3	α-Hydroxyisobutyric acid	2TMS	10.51	1140.8	131	NIST (877)
4	RI1142 unknown		10.53	1141.7	238	
5	Glyoxylic acid oxime	2TMS	10.6	1144.5	218	NIST (783)
6	Sarcosine	2TMS	10.73	1150.3	218	NIST (933)
7	RI1157 unknown		10.89	1157.1	231	
8	RI1158 unknown		10.92	1158.2	177	
9	2-Furancarboxylic acid	1TMS	10.94	1159.1	125	NIST, GMD (887)
10	RI1162 unknown		11.01	1161.9	234	
11	RI1164 unknown		11.06	1164.3	257	
12	2-Hydroxybutanoic acid		11.24	1171.6	233	GMD (776)
13	4-Dimethylaminobutyric acid		11.25	1172.5	143	NIST (817)
14	RI1176 unknown		11.35	1176.2	128	
15	RI1180 unknown		11.43	1179.7	216	

16	2-Aminobutyric acid		11.48	1181.7	204	NIST (881)
17	RI1186 unknown		11.57	1185.8	322	
18	Methylphosphate	2TMS	11.69	1190.8	256	NIST, GMD (914)
19	3-Aminoisobutyric acid		11.92	1200.5	130	NIST (817)
20	RI1201 unknown		11.93	1200.9	159	
21	RI1205 unknown		12.05	1205.3	188	
22	RI1216 unknown		12.33	1216.1	116	
23	RI1217 unknown		12.34	1216.6	169	
24	Valine	2TMS	12.43	1220.1	246	NIST (827), ASL
25	RI1225 amine		12.56	1224.9	202	
26	RI1227 unknown		12.6	1226.6	227	
27	RI1228 unknown		12.62	1227.6	266	
28	2-Phenylethanol	1TMS	12.81	1234.8	179	NIST (889)
29	2-Hydroxyisocaproic acid		12.82	1235.2	103	NIST (926)
30	2-Hydroxy-3-methylvaleric acid	1TMS	12.89	1237.8	159	NIST (785)
31	Diethylenglycol	2TMS	13.11	1246.4	117	NIST (713)
32	Benzoic acid	1TMS	13.33	1254.8	179	NIST, GMD (904)
33	Ethanolamine	3TMS	13.5	1261.6	174	NIST (923)
34	Serine peak 1	2TMS	13.55	1263.2	116	NIST (722), ASL
35	Leucine	2TMS	13.68	1268.5	232	NIST (859)
36	Glycerol	3TMS	13.82	1273.6	293	NIST (830), ASL
37	Phosphoric acid	3TMS	13.86	1275.5	314	NIST (900), ASL
38	RI1280 unknown		13.98	1279.9	280	
39	RI1285 unknown		14.11	1285	204	
40	Isoleucine	2TMS	14.21	1289	232	NIST (846), ASL
41	Threonine peak 1	2TMS	14.28	1291.8	130	NIST (826), ASL
42	Proline	2TMS	14.31	1292.7	244	NIST (813), ASL
43	Glycine peak 2	3TMS	14.44	1297.9	276	NIST (864), ASL
44	Nicotinic acid	1TMS	14.5	1300.2	180	NIST (861), ASL
45	RI1301 unknown		14.52	1300.7	239	
46	RI1302 organic acid	2TMS	14.56	1302.2	156	NIST, GMD (923)
47	RI1304 unknown		14.6	1303.7	294	
48	Succinic acid	2TMS	14.72	1308.3	262	GMD (750), ASL
49	RI1312 unknown		14.82	1312.2	315	
50	Glyceric acid	3TMS	14.94	1316.8	292	NIST (942), ASL
51	Uracil	2TMS	15.19	1326.4	241	NIST (898)

52	3,6-Dimethylpiperazine-2,5-dione		15.34	1332.4	271	GMD (807)
53	Fumaric acid	2TMS	15.43	1335.8	245	NIST (938), ASL
54	Alanine peak 2	3TMS	15.53	1339.7	262	NIST (700), ASL
55	Serine peak 2	3TMS	15.6	1342.2	306	NIST (805), ASL
56	2,5-Hydroxypyrazine		15.61	1342.7	241	NIST (877)
57	Pipecolic acid	2TMS	15.68	1345.4	156	NIST, GMD (848)
58	RI1354 unknown		15.91	1354	117	
59	RI1355 unknown		15.93	1355	164	
60	RI1356 unknown		15.96	1355.9	141	
61	Serine methyl ester	3TMS	16.05	1359.6	232	GMD (788)
62	Threonine peak 2	3TMS	16.16	1363.8	320	NIST (928), ASL
63	RI1378 unknown		16.54	1378.3	154	
64	RI1379 unknown		16.56	1379	342	
65	RI1384 unknown		16.69	1384.1	373	
66	RI1386 Amino base deriv.	2TMS	16.74	1386.1	270	NIST (675)
67	S-Methyl cysteine	2TMS	16.94	1393.7	162	NIST, GMD (880)
68	β-alanine	3TMS	16.99	1395.8	248	NIST, GMD (845), ASL
69	RI1399 unknown		17.09	1399.3	540	
70	Aspartic acid peak 1	2TMS	17.11	1400.4	160	NIST, GMD (750), ASL
71	RI1406 unknown		17.24	1406.4	320	
72	RI1410 unknown		17.32	1410.4	540	
73	RI1414 unknown		17.4	1414.3	247	
74	Homoserine	3TMS	17.46	1417.4	218	NIST, GMD (800), ASL
75	RI1420 unknown		17.51	1419.8	350	
76	RI1427 unknown		17.61	1424.6	299	
77	Ornithine-1,5-lactam	2TMS	17.7	1429.2	258	NIST, GMD (912)
78	RI1431 unknown		17.73	1430.7	232	
79	RI1434 unknown		17.8	1433.9	252	
80	Aminomalonic acid	3TMS	17.92	1439.8	320	NIST, GMD (882)
81	RI1448 unknown		18.09	1448.3	155	
82	Thiazolidine-4-carboxylic acid	2TMS	18.11	1449.1	160	NIST (762)
83	RI1455 unknown		18.23	1455	285	
84	Malic acid	3TMS	18.3	1458.4	245	NIST (930), ASL
85	RI1462 unknown		18.38	1462.4	275	
86	RI1466 unknown		18.46	1466.5	128	
87	RI1471 unknown		18.56	1471.1	186	
88	Nicotinamide	1TMS	18.62	1474	179	NIST, GMD (852)

89	Erythritol	4TMS	18.64	1475	217	NIST (890), ASL
90	RI1478 unknown		18.69	1477.8	112	
91	Asparagine-H2O	2TMS	18.75	1480	243	GMD (880)
92	RI1483 unknown		18.81	1483.5	188	
93	RI1486 unknown		18.86	1485.9	188	
94	Aspartic acid peak 2	3TMS	18.92	1488.7	202	NIST (829), ASL
95	Methionine	2TMS	18.94	1490.1	293	NIST, GMD (770), ASL
96	Aspartic acid peak 3	3TMS	19.04	1494.9	202	NIST (811)
97	Pyroglutamic acid	2TMS	19.21	1503.3	273	NIST (816), ASL
98	Glutamic acid peak 1	2TMS	19.29	1507.9	276	NIST (858), ASL
99	Ditertbutylphenol	1TMS	19.35	1510.9	263	NIST (703)
100	RI1514 unknown		19.4	1513.9	348	
101	RI1519 unknown		19.49	1518.9	260	
102	Cysteine	3TMS	19.62	1525.9	220	NIST, GMD (939)
103	Mevalonic acid	3TMS	19.66	1527.7	233	NIST (850)
104	2-Hydroxyglutaric acid	3TMS	19.67	1528.4	129	NIST, GMD (797), ASL
105	RI1541 unknown		19.9	1540.8	217	
106	4-Hydroxyphenylethanol	2TMS	19.98	1545.1	282	NIST, GMD (896)
107	RI1547 unknown		20.02	1547.3	202	
108	α-hydroxyglutaric acid	3TMS	20.03	1548.1	247	NIST (869), ASL
109	RI1553 unknown		20.13	1553.5	318	
110	α-ketoglutaric acid	1MEOX, 2TMS	20.14	1554	198	NIST (700), ASL
111	2-Phenyllactic acid	2TMS	20.22	1558.3	193	NIST, GMD (746)
112	RI1560 unknown		20.25	1559.8	186	
113	Asparagine peak 1	4TMS	20.5	1573.5	188	NIST, GMD (788)
114	β-hydroxymethylglutaric acid	3TMS	20.53	1575.3	247	NIST (676)
115	Ornithine peak 1	3TMS	20.79	1589.5	244	NIST, GMD (739)
116	Glutamic acid peak 2	3TMS	21	1600.9	363	NIST (833), ASL
117	Phenylalanine	2TMS	21.07	1604.8	266	NIST (883), ASL
118	RI1612 organic acid		21.2	1612.4	229	GMD (625)
119	RI1613 unknown		21.22	1613.3	258	
120	RI1620 unknown		21.34	1620.2	254	
121	4-Hydroxyphenylacetic acid	3TMS	21.36	1621.3	179	NIST, GMD (785)
122	RI1627 unknown		21.46	1627.3	258	

123	Lauric acid	1TMS	21.55	1632.1	257	NIST, GMD (725)
124	RI1636 unknown		21.62	1636	360	
125	RI1638 unknown		21.65	1637.8	346	
126	Homocysteine	3TMS	21.68	1639.7	234	NIST, GMD (872)
127	RI1642 unknown		21.72	1641.6	275	
128	RI1643 unknown		21.74	1642.8	290	
129	Phloroglucinol	3TMS	21.77	1644.7	342	NIST (700), ASL
130	Asparagine peak 2	3TMS	21.88	1651.1	348	NIST, GMD (937)
131	Ribose	1MEOX, 4TMS	21.91	1652.4	103	NIST (712), ASL
132	RI1655 unknown		21.95	1654.9	320	
133	Cysteinesulfinic acid	3TMS	22.04	1659.8	252	GMD (738)
134	RI1662 unknown		22.08	1662.4	243	
135	RI1666 unknown		22.15	1666.2	282	
136	RI1671 unknown		22.24	1671.2	326	
137	RI1676 unknown		22.32	1675.7	275	
138	RI1678 pentitol		22.36	1678.5	217	
139	RI1681 unknown		22.4	1680.6	319	
140	Lysine peak 1	3TMS	22.47	1684.5	362	NIST, GMD (833)
141	RI1686 unknown		22.49	1685.8	199	
142	RI1688 unknown		22.53	1687.6	218	
143	RI1689 unknown		22.55	1688.9	116	
144	α-aminoadipic acid	3TMS	22.63	1693.8	362	NIST, GMD (880)
145	Arabitol	5TMS	22.7	1697.5	307	NIST (832)
146	Glycerol-2-phosphate	4TMS	22.81	1704	243	NIST (893)
147	RI1708 unknown		22.88	1708	331	
148	RI1709 unknown		22.89	1708.6	304	
149	Putrescine	4TMS	22.92	1710.2	174	NIST (856), ASL
150	2-Aminopimelic acid	3TMS	23.01	1715.9	376	NIST (796)
151	Orotic acid	3TMS	23.18	1725.4	372	NIST (921)
152	Arginine deriv. peak 1	2TMS	23.27	1730.6	184	GMD (712)
153	RI1741 unknown		23.44	1741	174	
154	Glycerol-3-phosphate	4TMS	23.48	1742.9	445	NIST (781), ASL
155	RI1747 unknown		23.55	1747.2	217	
156	RI1752 unknown organic acid		23.64	1752.5	129	
157	4,5-Dihydroorotic acid	3TMS	23.67	1754.4	359	NIST, GMD (809)
158	Glutamine	3TMS	23.73	1757.4	245	NIST (888)
159	RI1760 unknown		23.78	1760.3	116	
160	Methionine sulfoxide	3TMS	23.85	1764.9	128	GMD (710)
161	RI1765 unknown		23.86	1765.2	175	
162	RI1766 unknown		23.88	1766.2	276	
163	RI1768 unknown		23.9	1767.6	376	

164	RI1769 unknown		23.93	1769.5	175	
165	RI1779 hydrocarbon		24.09	1778.6	97	
166	RI1780 unknown		24.12	1780.3	317	
167	RI1782 sugar		24.15	1782.1	204	
168	RI1784 unknown		24.19	1784.3	595	
169	RI1788 unknown		24.25	1788.2	301	
170	Glyceric acid-3-phosphate	4TMS	24.29	1790.6	357	NIST (831), ASL
171	RI1793 phosphate		24.33	1792.7	473	
172	Ornithine peak 2	4TMS	24.38	1795.5	420	NIST (851), ASL
173	Citric acid	4TMS	24.45	1799.4	257	NIST (929), ASL
174	Isocitric acid	4TMS	24.49	1802.3	245	NIST (736)
175	Hypoxanthine	2TMS	24.51	1803.3	280	NIST, GMD (834)
176	Arginine deriv. peak 2	3TMS	24.58	1807.5	373	NIST, GMD (849)
177	RI1808 polyol	5TMS	24.58	1808	231	NIST (608)
178	RI1814 unknown		24.69	1814.5	218	
179	2-Methylcitric acid	4TMS	24.71	1815.4	287	NIST, GMD (715)
180	RI1820 organic acid		24.79	1820.4	247	
181	RI1824 unknown		24.85	1824	116	
182	RI1826 hexose		24.88	1826.1	103	
183	Myristic acid	1TMS	25	1833.5	285	NIST (775)
184	Lysine peak 2	3TMS	25.07	1837.6	362	NIST, GMD (788)
185	RI1839 unknown		25.09	1839.1	245	
186	RI1847 hexose		25.22	1846.7	307	
187	RI1853 sugar		25.31	1852.6	205	
188	Fructose	1MEOX, 5TMS	25.32	1853.1	103	
189	3-Hydroxyanthranillic acid	3TMS	25.32	1853.2	354	NIST (853)
190	Adenine	2TMS	25.46	1861.6	279	NIST (879)
191	RI1875 unknown		25.67	1874.6	204	
192	Galactose	1MEOX, 5TMS	25.68	1875	319	NIST (787), ASL
193	Tyrosine peak 1	2TMS	25.7	1875.9	208	NIST (829), ASL
194	Glucose	1MEOX, 5TMS	25.77	1880.2	319	
195	RI1881 unknown		25.78	1881.2	202	
196	RI1882 unknown		25.79	1881.6	216	
197	RI1891 unknown		25.94	1891	142	
198	Lysine peak 3	4TMS	26.17	1905.2	434	NIST (844), ASL
199	Histidine	3TMS	26.23	1908.8	356	NIST, GMD (771)
200	Mannitol	6TMS	26.28	1911.8	421	NIST (869), ASL

201	Tyrosine peak 2	3TMS	26.5	1925.7	354	NIST (892), ASL
202	Vanillylmandelic acid	3TMS	26.54	1928.4	297	NIST, GMD (601)
203	N-Carbamoyl aspartic acid		26.54	1928.7	160	GMD (634)
204	Ononitol	5TMS	26.61	1932.9	318	NIST, GMD (791)
205	Pentadecanoic acid	1TMS	26.63	1934.3	117	NIST, GMD (602), ASL
206	RI1937 unknown		26.67	1936.8	204	
207	RI1938 unknown		26.7	1938.5	300	
208	RI1943 unknown		26.79	1943.5	260	
209	RI1946 unknown		26.82	1946.1	211	
210	RI1950 unknown		26.87	1949.6	230	
211	Serine-Leucine	2TMS	26.94	1953.8	332	NIST (538)
212	RI1959 unknown		27.03	1959.2	142	
213	RI1962 unknown		27.07	1961.7	149	
214	RI1964 hexose		27.11	1964.4	204	
215	RI1966 unknown		27.14	1965.8	218	
216	RI1968 unknown		27.17	1968	415	
217	RI1970 unknown		27.2	1969.9	158	
218	RI1975 unknown		27.27	1974.9	300	
219	Pantothenic acid	3TMS	27.3	1976.4	291	NIST, GMD (894)
220	Heptadecanol		27.4	1982.6	83	NIST (621)
221	N-Acetyl ornithine	3TMS	27.42	1984.3	174	GMD (662)
222	RI1985 sugar		27.44	1985.5	204	
223	RI1986 amine		27.45	1986	230	
224	RI1988 unknown		27.48	1988.2	293	
225	RI1989 unknown		27.5	1988.8	358	
226	RI1990 unknown		27.51	1990	214	
227	RI1998 unknown		27.64	1998.2	320	
228	RI2003 unknown		27.72	2003	204	
229	RI2006 phosphate		27.76	2005.8	243	
230	Scillo-Inositol	6TMS	27.77	2006.7	318	NIST (776)
231	Palmitelaidic acid	1TMS	27.84	2011.3	145	NIST, GMD (926), ASL
232	RI2031 unknown		28.15	2031.3	142	
233	Palmitic acid	1TMS	28.16	2032.1	328	NIST, GMD (937), ASL
234	RI2033 unknown		28.17	2033	218	
235	RI2041 unknown		28.29	2040.8	291	
236	RI2042 unknown		28.3	2041.7	361	
237	RI2044 amine		28.33	2043.6	174	
238	N-Acetylglucosamine	4TMS	28.65	2064.3	274	GMD (414)
239	Myo-Inositol	6TMS	28.73	2069.4	432	NIST (883), ASL
240	RI2084 pentose phosphate		28.95	2084.1	315	
241	RI2085 unknown		28.96	2084.8	264	
242	RI2085 sugar		28.97	2085.5	307	
243	Ribose-5-phosphate	1MEOX, 5TMS	29.04	2089.9	315	NIST (811)

244	N-Acetyl lysine		29.11	2095.1	174	NIST (810)
245	RI2097 unknown		29.15	2097.2	349	
246	RI2102 unknown		29.21	2101.7	307	
247	RI2106 unknown		29.28	2106.1	325	
248	RI2109 sugar		29.32	2109.1	319	
249	RI2114 sugar		29.4	2114.4	319	
250	N-Acetyl tyrosine	2TMS	29.5	2121.4	179	NIST, GMD (847)
251	Margaric acid	1TMS	29.64	2130.7	327	NIST (732)
252	RI2133 unknown		29.68	2132.8	353	
253	Octadecanol	1TMS	29.79	2140.4	327	NIST (624)
254	RI2145 unknown		29.86	2145	420	
255	RI2154 unknown		29.99	2154	174	
256	Kynurenine peak 1	2TMS	30.08	2159.9	192	NIST, GMD (787)
257	RI2164 unknown		30.14	2164.4	213	
258	RI2167 unknown		30.19	2167.3	265	
259	RI2173 unknown		30.28	2173.2	354	
260	Spermidine	4TMS	30.3	2174.9	174	NIST, GMD (794)
261	Kynurenine peak 2	3TMS	30.38	2180	307	NIST, GMD (736)
262	RI2185 unknown		30.45	2185.1	130	
263	RI2194 phosphate		30.59	2194.4	342	
264	Tryptophan	2TMS	30.64	2198.1	202	NIST, GMD (810), ASL
265	Oleic acid	1TMS	30.69	2201	339	NIST, GMD (808), ASL
266	RI2203 carbohydrate		30.72	2203.2	319	
267	RI2210 unknown		30.82	2210.2	381	
268	RI2223 unknown		31.01	2223.5	253	
269	Stearic acid	1TMS	31.06	2227.4	356	NIST (876), ASL
270	RI2233 carbohydrate		31.15	2233.5	204	
271	Quinaldic acid	3TMS	31.35	2247.3	406	NIST, GMD (677)
272	RI2266 unknown		31.61	2265.6	293	
273	RI2268 unknown		31.64	2267.6	309	
274	RI2270 unknown		31.67	2270	332	
275	RI2271 unknown		31.69	2271.4	466	
276	RI2272 unknown		31.71	2272.4	204	
277	RI2280 glycoside	4TMS	31.81	2279.8	204	NIST (712), ASL
278	RI2295 disaccharide		32.03	2294.7	361	
279	RI2313 unknown		32.28	2313.1	357	
280	RI2316 unknown		32.33	2316.3	334	
281	RI2333 unknown		32.56	2333.2	393	
282	RI2338 unknown		32.62	2338	204	
283	RI2342 unknown		32.68	2342	344	
284	RI2360 unknown		32.92	2359.8	318	
285	Myristoylglycerol	2TMS	33.11	2373.4	343	NIST (647)

286	Myo-Inositol-1-phosphate	7TMS	33.25	2383.3	318	NIST, GMD (900)
287	Uridine peak 1	3TMS	33.3	2387.1	259	NIST, GMD (769)
288	RI2399 unknown		33.46	2399.1	285	
289	Eicosanoic acid	1TMS	33.74	2420.1	369	NIST, GMD (740)
290	RI2424 amino base deriv.	3TMS	33.79	2424.2	329	NIST (573)
291	Uridine peak 2	3TMS	34.07	2445	169	NIST, GMD (883)
292	RI2467 unknown		34.36	2466.8	357	
293	RI2468 unknown		34.37	2467.9	339	
294	RI2483 unknown		34.57	2482.8	273	
295	RI2495 unknown		34.74	2495.2	553	
296	2-Palmitoylglycerol	2TMS	35.16	2528.1	129	NIST (701)
297	RI2544 unknown		35.36	2544.2	369	
298	Inosine	4TMS	35.5	2554.7	348	NIST, GMD (900)
299	1-Palmitoylglycerol	2TMS	35.57	2560.7	239	NIST, GMD (919)
300	RI2575 disaccharide	8TMS	35.76	2575.5	361	NIST (772), ASL
301	Adenosine peak 1	3TMS	35.86	2583.4	468	NIST, GMD (936)
302	RI2595 disaccharide		36.02	2595.4	361	
303	Adenosine peak 2	4TMS	36.22	2611.6	236	NIST, GMD (832)
304	RI 2646 disaccharide	8TMS	36.63	2645.6	361	NIST, GMD (849), ASL
305	Glycerol-1-margaric acid	2TMS	36.72	2653	385	NIST (587)
306	RI 2656 disaccharide	8TMS	36.77	2656.4	361	NIST, GMD (598), ASL
307	Trehalose	8TMS	37.28	2698.4	451	NIST, GMD (885)
308	2-Stearoylglycerol	2TMS	37.45	2712.6	341	NIST (807)
309	1-Oleoylglycerol	2TMS	37.59	2734.8	397	NIST, GMD (727)
310	RI2732 unknown		37.68	2731.7	175	
311	1-Stearoylglycerol	2TMS	37.89	2749.6	487	NIST, GMD (741)
312	RI2753 carbohydrate		37.93	2752.7	204	
313	RI2760 disaccharide		38.02	2760.2	204	
314	5-Methylthioadenosine	3TMS	38.13	2769.6	236	GMD (780)
315	RI2803 carbohydrate		38.53	2803.1	204	
316	Isomaltose	1MEOX, 8TMS	38.77	2824.4	361	NIST (710)
317	RI2830 disaccharide		38.84	2830.5	361	
318	RI2866 disaccharide		39.25	2865.9	361	
319	Hit Galactinol	9TMS	39.35	2875	204	NIST (823)
320	1-Eicosanoylglycerol	2TMS	40.02	2934.5	427	NIST (639)
321	RI2951 carbohydrate		40.21	2951.4	204	
322	RI2953 unknown		40.23	2953.3	583	

323	RI2970 unknown		40.41	2970.1	498	
324	RI2976 disaccharide		40.48	2976.2	361	
325	1-Methylinosine	3TMS	40.55	2981.9	259	NIST (758)
326	RI2994 disaccharide		40.68	2994.2	361	
327	RI3029 unknown		41.06	3029.1	291	
328	RI3050 disaccharide		41.29	3050.5	361	
329	RI3078 unknown		41.58	3077.6	259	
330	RI3079 isoprenoid		41.87	3104.5	81	
331	RI3119 unknown		42.03	3119.3	455	
332	RI3142 unknown		42.27	3142.4	353	
333	RI3165 sterol		42.5	3165	251	NIST (697)
334	RI3171 unknown		42.57	3171.2	133	
335	RI3195 unknown		42.81	3194.7	266	
336	Ergosterol	1TMS	42.97	3209.7	337	NIST (828)
337	RI3218 unknown		43.06	3218.5	133	
338	RI3228 sterol		43.13	3225.7	343	
339	RI3227 Ergosterol deriv.		43.15	3227.2	378	
340	RI3232 sterol		43.2	3232.4	466	
341	RI3256 unknown		43.43	3255.6	394	
342	RI3272 ergosterol deriv.		43.6	3271.7	365	
343	RI3282 ergosterol deriv.		43.7	3281.6	343	NIST (753)
344	RI3289 ergosterol deriv.		43.77	3289.1	472	NIST (746)
345	RI3297 trisaccharide	11TMS	43.85	3296.7	361	NIST (752)
346	Lanosterol	1TMS	43.99	3310.7	393	NIST, GMD (858)
347	RI3323 sterol		44.11	3322.7	482	
348	RI3326 trisaccharide	11TMS	44.14	3325.9	361	NIST (811)
349	RI3333 cholesterol deriv.		44.21	3332.8	484	NIST (814)
350	RI3357 unknown		44.45	3357	441	
351	RI3362 unknown		44.5	3361.8	561	
352	RI3363 sugar		44.89	3401.9	361	
353	RI3439 unknown		45.25	3439.2	129	
354	RI3449 trisaccharide	11TMS	45.35	3449.4	204	NIST (810)
355	RI3463 trisaccharide		45.48	3462.9	361	
356	RI3531 unknown		46.16	3531.3	648	
357	RI3564 trisaccharide		46.49	3563.9	361	
358	RI3608 unknown		46.96	3607.6	357	
359	RI3678 unknown		47.77	3677.8	129	
360	RI3730 unknown		48.44	3729.6	604	
361	RI3746 unknown		48.66	3745.9	383	
362	RI3749 unknown		48.7	3748.9	385	
363	RI3772 unknown		49.02	3771.9	383	
364	RI3777 unknown		49.08	3776.7	624	
365	RI3815 unknown		49.63	3814.8	371	
366	RI3820 unknown		49.72	3820.3	399	
367	RI3886 unknown		50.78	3886.5	383	
368	RI3890 unknown		50.83	3889.9	385	
369	RI3896 unknown		50.93	3896.1	413	
370	RI3902 unknown		51.04	3902.5	654	
371	RI3916 unknown		51.29	3915.8	383	
372	RI3919 unknown		51.36	3919.5	385	
373	RI3932 unknown		51.61	3932.2	371	

374	RI3938 unknown	51.73	3938.5	431	
375	RI3962 unknown	52.17	3961.8	399	
376	RI4030 unknown	53.48	4030.4	411	
377	RI4046 unknown	53.77	4046.1	129	
378	RI4064 unknown	54.12	4064.2	129	
379	RI4109 unknown	54.97	4108.7	399	NIST (786), ASL

RT – Retention time, min; **RI** – Kovach Retention Index calculated from the retention times of the alkanes.
a – Unidentified metabolites are labeled as "unknown" with an associated retention index (RI). The names of metabolites annotated only to a specific chemical class (for which the exact structure could not be assigned) begin with the RI followed by the name of the annotated chemical class. Annotation to specific chemical classes was confirmed by the presence of characteristic signals in the EI spectra. The metabolites are listed in order of increasing retention time (RT).
b – Numbers of the trimethylsilyl (TMS) and methyloxime (MEOX) groups in the structures of the annotated derivatives.
c – EI-MS libraries where metabolite EI mass spectra and RI were used for metabolite annotation: ASL – in-house RI and spectral library of authentic standards, NIST – National Institute of Standards and Technology, GMD – Golm Metabolome Database (http://gmd.mpimp-golm.mpg.de/). The number represents the NIST search match factor of the corresponding library.

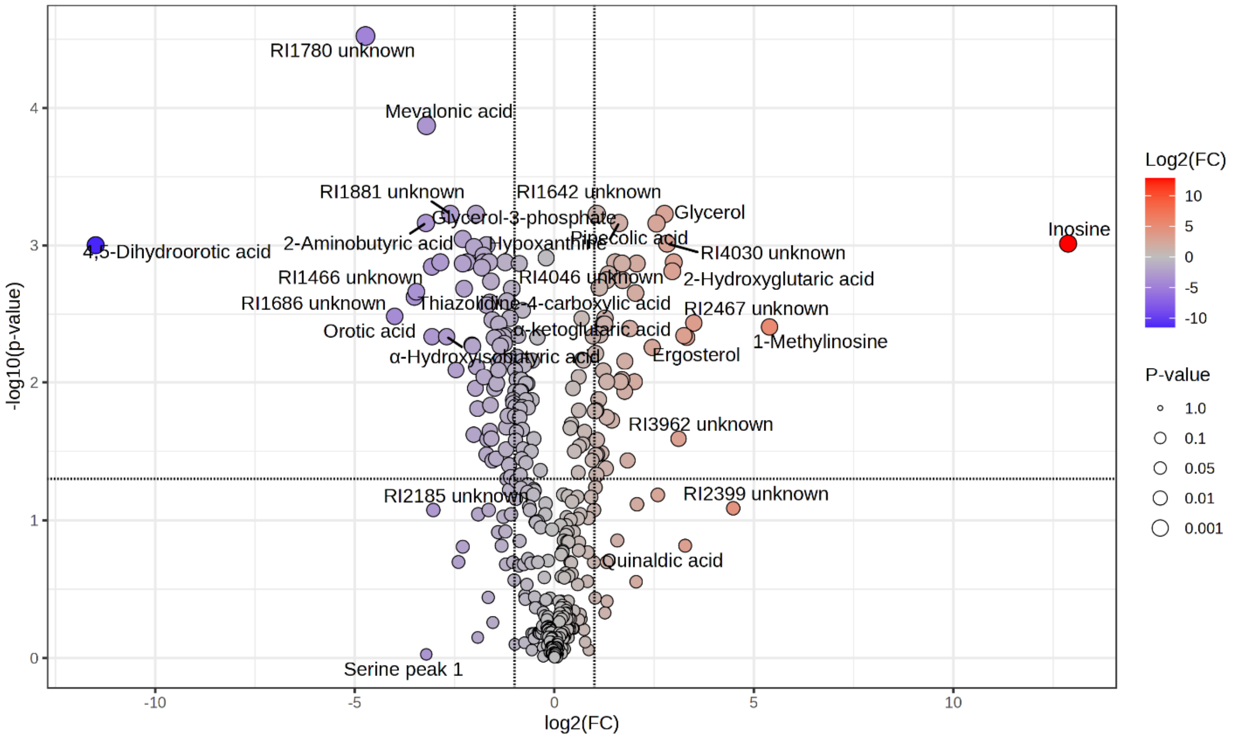

Figure S1. Volcano plot showing the metabolites contributing to the *ade12*-dependent changes in the metabolite profiles of *Saccharomyces cerevisiae*. The plot includes compounds with significantly ($p < 0.05$, FDR-adjusted) different abundances in the tested yeast strains (WT vs *ade12*) with fold changes ≥ 2. Data analysis was performed using MetaboAnalyst 6.0 (https://www.metaboanalyst.ca, accessed on 27.01.2025).

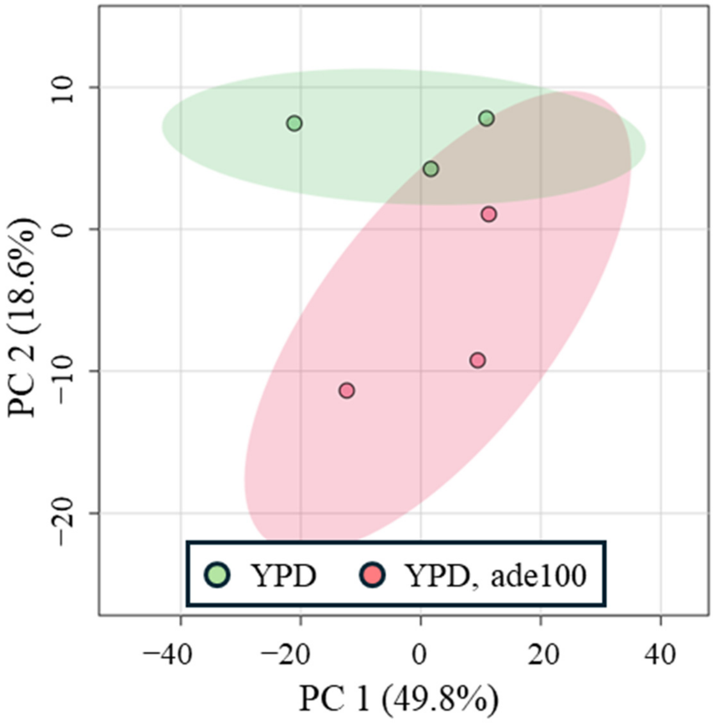

Figure S2. Sample scores for the first two principal components derived from PCA of the low molecular weight metabolite profiles of the wild type *S. cerevisiae* cells cultivated on pure YPD medium and YPD medium containing additional adenine (100 µg/ml).

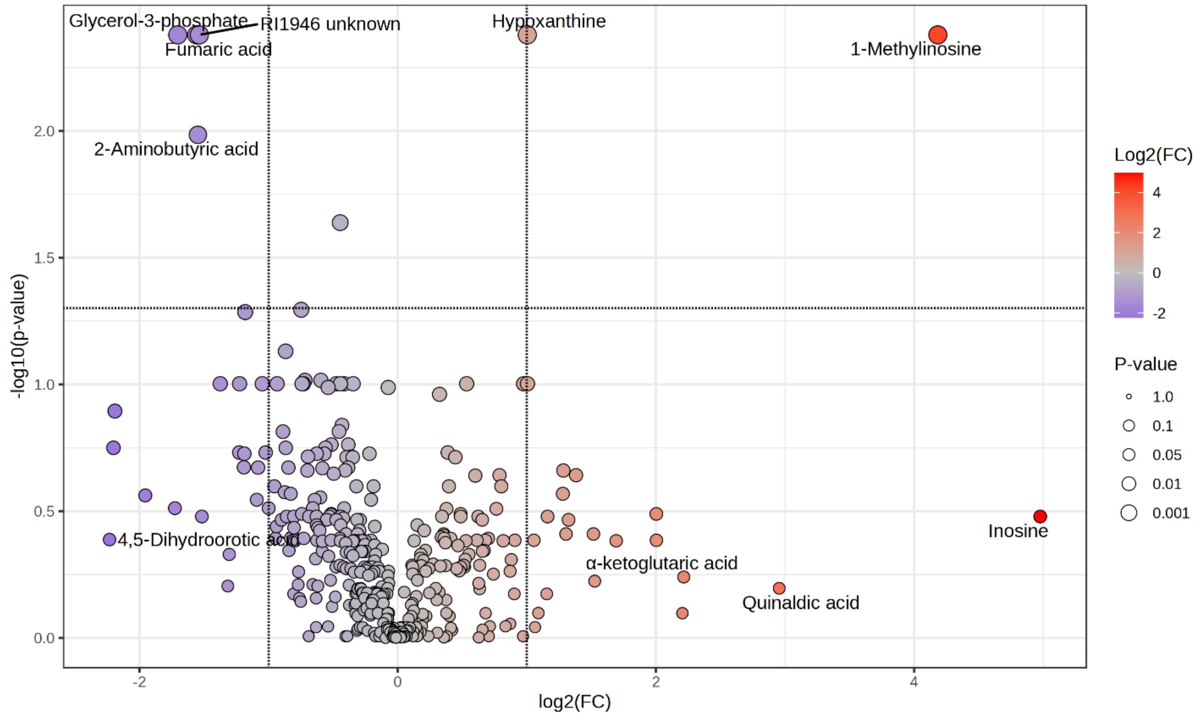

Figure S3. Volcano plot showing the metabolites contributing to the *ade12*-dependent changes in the metabolite profiles of *Saccharomyces cerevisiae* grown in the YPD medium supplemented with 100 µg/ml adenine. The plot includes compounds with significantly ($p < 0.05$, FDR-adjusted) different abundances in the tested yeast strains (WT *vs ade12*) with fold changes ≥ 2. Data analysis was performed using MetaboAnalyst 6.0 (https://www.metaboanalyst.ca, accessed on 27.01.2025).

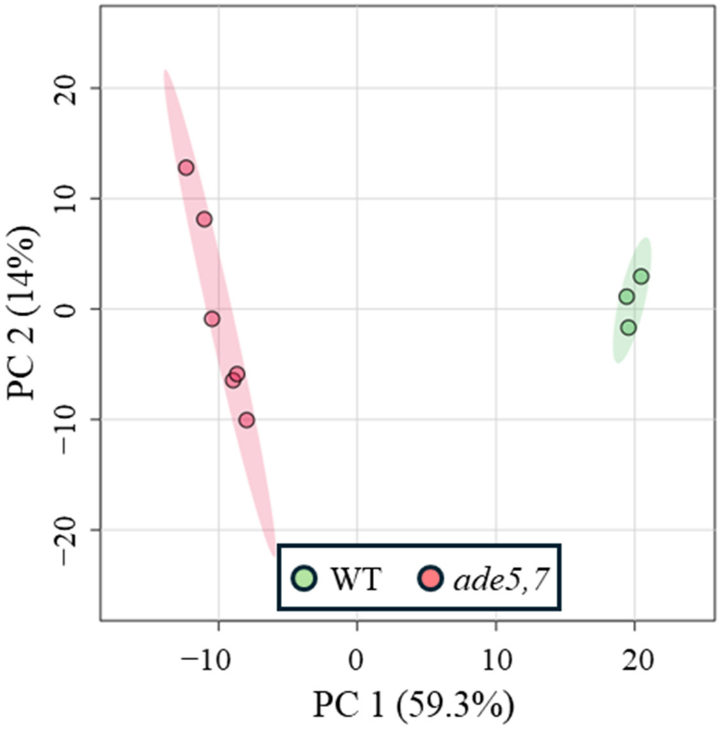

Figure S4. Sample scores for the first two principal components derived from PCA of the low molecular weight metabolite profiles of the *S. cerevisiae* cells (wild type and *ade5,7*) cultivated on YPD medium.

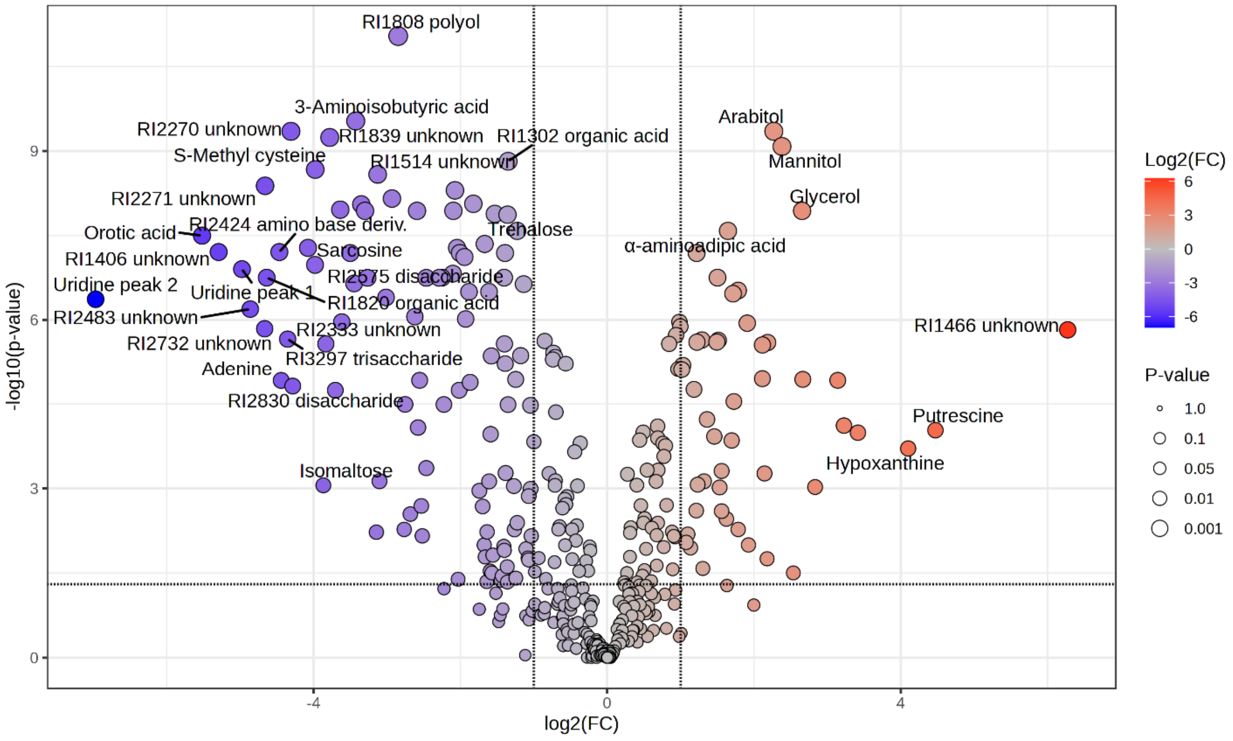

Figure S5. Volcano plot showing the metabolites contributing to the *ade12*-dependent changes in the metabolite profiles of *Saccharomyces cerevisiae* cells bearing the background *ade5,7* mutation. The plot includes compounds with significantly ($p < 0.05$, FDR-adjusted) different abundances in the tested yeast strains (*ade5,7 vs ade5,7ade12*) with fold changes ≥ 2. Data analysis was performed using MetaboAnalyst 6.0 (https://www.metaboanalyst.ca, accessed on 27.01.2025).

Table S4. The metabolites contributing to the *ade12*-dependent changes in the metabolite profiles of *Saccharomyces cerevisiae* cells bearing the background *ade5,7* mutation. The table includes identified or structurally annotated compounds having significantly ($p < 0.05$, FDR-adjusted) differing abundances in the tested yeast strains (*ade5,7* vs *ade5,7ade12*) with fold changes ≥ 2. The metabolites are grouped based on their chemical classes. The statistical analysis was performed using MetaboAnalyst 6.0 (http://www.metaboanalyst.ca, accessed on 27.01.2025).

Metabolite	Fold change	Adjusted *p*-value
**Down-regulated metabolites**		
Amino acids:		
Orotic acid	45.9	< 0.001
Sarcosine	16.9	< 0.001
S-Methyl cysteine	15.8	< 0.001
3-Aminoisobutyric acid	10.8	< 0.001
2-Aminobutyric acid	4.3	< 0.001
Threonine	4.1	0.040
Thiazolidine-4-carboxylic acid	3.7	< 0.001
Pipecolic acid	3.2	< 0.001
Cysteine	3.0	< 0.001
β-alanine	2.9	0.015
Kynurenine	2.7	0.035
Serine methyl ester	2.6	< 0.001
Glutamic acid	2.6	< 0.001
N-Acetyl tyrosine	2.3	0.004
Proline	2.2	< 0.001
Pantothenic acid	2.1	< 0.001
Cysteinesulfinic acid	2.0	0.007
Sugars, sugar-phosphates, and polyols:		
RI3297 trisaccharide	20.4	< 0.001
RI2830 disaccharide	19.5	< 0.001
RI2575 disaccharide	15.8	< 0.001
Isomaltose	14.6	< 0.001
RI2085 sugar	11.3	< 0.001
Glycerol-3-phosphate	10.2	< 0.001
RI2866 disaccharide	9.8	< 0.001
RI3564 trisaccharide	8.1	< 0.001
RI1808 polyol	7.2	< 0.001
RI2595 disaccharide	6.4	0.003
RI1853 sugar	6.0	< 0.001
Fructose	4.8	< 0.001
Ribose-5-phosphate	3.6	< 0.001
Trehalose	2.6	< 0.001
RI3050 disaccharide	2.2	0.012
RI2994 disaccharide	2.1	0.017
RI2976 disaccharide	2.1	0.018
Organic acids:		
RI1820 organic acid	24.9	< 0.001
2-Hydroxy-3-methylvaleric acid	3.8	< 0.001
2-Hydroxyisocaproic acid	2.6	< 0.001
2-Hydroxybutanoic acid	2.6	< 0.001
RI1302 organic acid	2.5	< 0.001
Nucleosides and nitrogenous bases:		
Uridine	125.3	< 0.001
RI2424 amino base derivative	22.1	< 0.001
Adenine	21.7	< 0.001
Adenosine	12.3	< 0.001

	Sterols and their derivatives:	
RI3272 ergosterol derivative	4.3	< 0.001
RI3282 ergosterol derivative	4.1	< 0.001
Lanosterol	4.0	< 0.001
RI3333 cholesterol derivative	3.2	0.010
RI3228 sterol	3.0	0.029
RI3289 ergosterol derivative	3.0	0.031
RI3227 ergosterol derivative	2.3	< 0.001
	Miscellaneous:	
Nicotinamide	5.5	< 0.001
2-Phenylethanol	2.6	0.046
	**Up-regulated metabolites**	
	Amino acids:	
Ornithine	10.7	< 0.001
Arginine derivative	9.4	< 0.001
Ornithine-1,5-lactam	7.1	< 0.001
Histidine	5.8	0.031
Glutamine	3.8	0.010
3-Hydroxyanthranillic acid	3.8	< 0.001
α-Aminoadipic acid	3.1	< 0.001
N-Acetyl ornithine	2.9	0.002
Methionine sulfoxide	2.4	< 0.001
Homoserine	2.3	< 0.001
	Sugars and polyols:	
RI1826 hexose	8.8	< 0.001
Glycerol	6.3	< 0.001
Mannitol	5.2	< 0.001
Arabitol	4.8	< 0.001
RI1847 hexose	3.2	< 0.001
RI2233 sugar	2.9	< 0.001
Glucose	2.7	< 0.001
Erythritol	2.6	< 0.001
RI1985 sugar	2.5	< 0.001
RI2114 sugar	2.4	< 0.001
	Organic acids:	
Glyceric acid	4.5	< 0.001
Mevalonic acid	3.3	< 0.001
α-ketoglutaric acid	2.1	0.009
α-hydroxyglutaric acid	2.0	< 0.001
	Amines:	
Putrescine	22.2	< 0.001
Spermidine	4.5	0.018
RI1986 amine	3.4	< 0.001
Ethanolamine	2.5	0.026
N-Acetylglucosamine	2.0	< 0.001
	Nitrogenous bases:	
Hypoxanthine	17.2	< 0.001
Uracil	3.3	< 0.001
